# Dual-Mode Colorimetric and Fluorescent Detection of Cu^2+^ Based on a Two-Component Sensing Platform Integrating an Imine Derivative and Rhodamine B

**DOI:** 10.1021/acsomega.6c07479

**Published:** 2026-09-23

**Authors:** Rosa M. Sevillano-Arredondo, Jorge Molina-González, Marlene Vargas-Zamarripa, Oracio Serrano, Gonzalo Ramírez-García

**Affiliations:** † Centro de Física Aplicada y Tecnología, 7180 Universidad Nacional Autónoma de México, Avanzada. 3001, Boulevard Juriquilla, Querétaro 76000, Mexico; ‡ División de Ciencias Naturales y Exactas, Departamento de Química, 14654 Universidad de Guanajuato, Cerro de la Venada s/n, Guanajuato 36050, Mexico

## Abstract

Copper is an essential trace element involved in key biological and environmental processes; however, its imbalance can lead to severe health and ecological consequences, making its accurate quantification crucial. In this work, a two-component colorimetric and fluorescent sensing platform based on the imine derivative 2-(((6-bromopyridin-2-yl)­methylene)­amino)-4-(*tert*-butyl)­phenol (BPTP) and rhodamine B (RhB) was developed for the selective detection of Cu^2+^ in aqueous media. The BPTP ligand, synthesized via a Schiff base condensation reaction and fully characterized by NMR spectroscopy, enables rapid and highly selective colorimetric recognition of Cu^2+^ at pH ≥ 7.0, producing a distinct visible transition from yellow to red associated with the formation of a 2:1 BPTP–Cu^2+^ complex. This binding mode was confirmed by UV–vis titration, ESI-MS analysis, and density functional theory (DFT) calculations, which also elucidate the structural and electronic changes responsible for the observed bathochromic shift. To enhance analytical performance, RhB was incorporated as an independent fluorescent reporter, generating a complementary optical channel governed by an inner filter effect (IFE) induced by the absorption band of the BPTP–Cu^2+^ complex. This dual-mode strategy significantly improves both sensitivity and selectivity in the presence of competing metal ions, achieving detection limits of 2.1 μM (colorimetric, BPTP), and 1.9 μM (fluorescence, RhB + BPTP). The platform was successfully applied to Cu^2+^ quantification in tap water, yielding concentrations within World Health Organization (WHO) limits. Overall, the RhB–BPTP platform represents a robust, sensitive, and cost-effective strategy for real-time copper monitoring in environmental samples.

## Graphical abstract

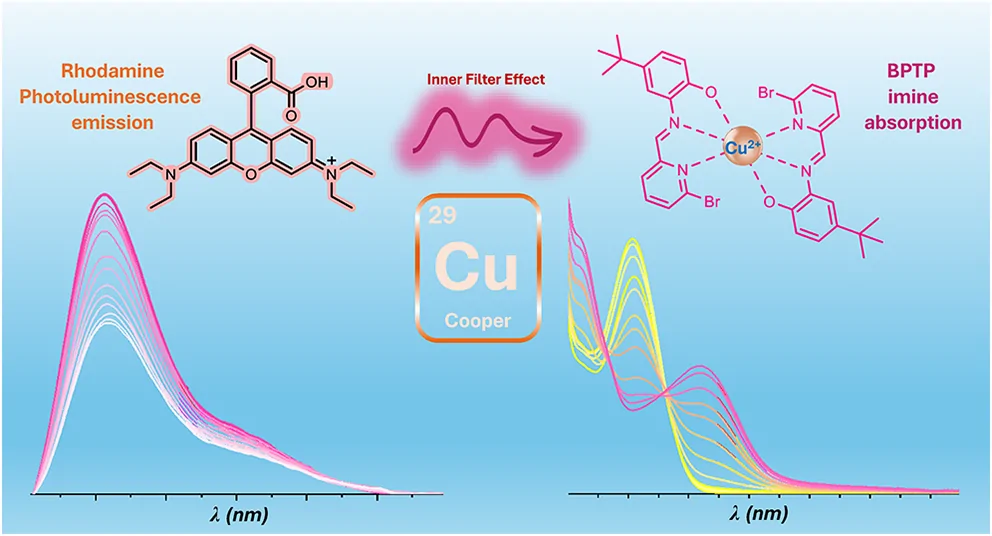

## Introduction

Copper is an essential trace element in the human body, is present in some important proteins, and therefore plays a crucial role in many biological processes, including cellular respiration, iron metabolism, and neurotransmission.^1–3^ However, excessive intake of copper is linked to neurodegenerative diseases such as Alzheimer’s, Parkinson’s, and Wilson’s diseases, while a deficit is related to anemia, colon cancer, and brittle bones.^4–7^ Copper is also an essential micronutrient for plants, fundamental for photosynthesis, respiration, protein synthesis, and plant structure. Its deficiency causes chlorosis and malformations in young leaves, whereas its excess is toxic to roots.^8,9^ The primary source of human copper exposure is diet, followed by drinking water.^10–12^ Consequently, the World Health Organization (WHO) and the Environmental Protection Agency (EPA) have established a strict limit of 1.3 mg/L for copper concentration in drinking water.^13^ For plants, the main source of absorption is naturally from the soil, although they also receive copper through fertilizers, pesticides, and irrigation water.^14^ All of the above make it necessary to develop reliable strategies for its quantification.

Traditional analytical techniques for Cu^2+^ detection, such as inductively coupled plasma mass spectrometry (ICP–MS), atomic absorption spectroscopy (AAS), and electrochemical methods, offer high sensitivity and low detection limits. Nevertheless, these methods are often constrained by the need for expensive instrumentation, complex sample preparation, and specialized personnel, making them unsuitable for *on-site*, real-time monitoring. In this context, colorimetric chemosensors have emerged as a powerful alternative for detecting copper ions, owing to their simplicity and cost-effectiveness, without the need for sophisticated equipment.^15–19^ Colorimetric chemosensors are particularly popular because they exhibit a color change upon interaction with copper ions that is readily observable to the naked eye.^20^ Among the various organic molecular structures used for colorimetric metal ion sensing, the imines or Schiff bases-compounds stand out for being a versatile coordination system.^21–24^ Schiff bases are characterized by the presence of an imine group (>C=N−) and provide a stable platform for forming chelate complexes with transition metals, often resulting in distinct color changes due to intramolecular charge transfer (ICT) mechanisms.^25–27^ Furthermore, their ease of synthesis and structural tunability allow for the optimization of selectivity and sensitivity toward specific analytes.

To enhance the practical applicability of these molecules, integrating fluorescent signaling provides a complementary and robust approach.^28^ Fluorescence has emerged as a powerful tool for sensing,^29^ since this approach monitors the quenching or intensification of emissions, usually resulting from the overlap of the chromophore’s absorption and the fluorophore’s emission bands. Fluorescence quenching by dynamic or static processes generally involves Förster resonance energy transfer (FRET), which is a highly efficient deactivation pathway. However, these systems are complex and limited by the distance between the donor and the acceptor.^30^ In contrast, fluorescence attenuation based on inner filter effect (IFE) provides a versatile platform for analyte detection that is nondependent on the proximity between the donor and the acceptor; besides, it does not require chemical bonding or direct conjugation between the chromophore and the fluorophore, which greatly simplifies the development of chemosensors.^28^ Therefore, fluorescence sensing based on the IFE mechanism has been extensively explored using a wide array of fluorophores, including photoluminescent nanomaterials and dye molecules.^31^ In addition, the combination of dual colorimetric and fluorescent mode detection represents a synergistic strategy that enhances sensing reliability, sensitivity, and selectivity, and the integration of these independent signaling pathways could improve the accuracy of copper ion detection.^32^

Thereby, in this work, the imine derivative 2-(((6-bromopyridin-2-yl)­methylene)­amino)-4-(*tert*-butyl)­phenol (BPTP) was synthesized and successfully characterized for the selective detection of Cu^2+^. The BPTP chemosensor exhibits a colorimetric response toward Cu^2+^ in water media with high selectivity and sensitivity at pH 7. The sensing mechanism, characterized by a rapid and distinct colorimetric response, was investigated using UV–vis spectrophotometry. Furthermore, by integrating RhB into the system, a dual-mode photoluminescent platform was developed. This combined approach significantly enhanced analytical sensitivity, yielding a lower limit of detection (LOD) compared to the individual colorimetric responses of both the free BPTP and the RhB–BPTP platform. In this case, steady-state and time-resolved fluorescence studies revealed that the RhB quenching mechanism corresponds to IFE effect. Finally, the practical utility of the BPTP sensor was validated by quantifying copper in tap water at concentrations relevant to the limits recommended by international regulatory agencies.

## Experimental Section

### Reagents

All reagents were of analytical grade and used without further purification unless otherwise stated. 6-Bromo-2-pyridinecarboxaldehyde, 2-amino-4-*tert*-butylphenol, NaCl, KCl, Ca­(NO_3_)_2_·4H_2_O, Mn­(CH_3_CO_2_)_2_, Co­(NO_3_)_2_·6H_2_O, Ni­(NO_3_)_2_·6H_2_O, Zn­(NO_3_)_2_·6H_2_O, Pb­(NO_3_)_2_, AlCl_3_, and Cd­(NO_3_)_2_·4H_2_O were purchased from Merck/Sigma-Aldrich. FeSO_4_·7H_2_O, HgCl_2_, CrCl_3_·3H_2_O, Cu­(NO_3_)_2_·3H_2_O, Mg­(NO_3_)_2_·6H_2_O, and absolute ethanol were obtained from Karal. Absolute ethanol (≥99.8%) was used as solvent without additional purification. All aqueous solutions were prepared using deionized water (resistivity ≥ 18.2 MΩ·cm at 25 °C). Stock solutions of metal salts (typically 1.0 × 10^–2^ M) were freshly prepared prior to use and diluted as required. Experimental measurements were carried out at room temperature (25 ± 2 °C) under ambient atmospheric conditions.

### Synthesis and Structural Characterization of 2-(((6-Bromopyridin-2-yl)­methylene)­amino)-4-(*tert*-butyl)­phenol (BPTP)

The Schiff base ligand was prepared according to our previously reported procedure.^33^ Briefly, equimolar mixture of 6-bromo-2-pyridinecarboxaldehyde (500 mg, 2.7 mmol) and 2-amino-4-*tert*-butylphenol (446 mg, 2.7 mmol) were stirred in ethanol (20 mL) at room temperature for 3 h. After solvent removal, the resulting yellow solid was recrystallized from ethanol to afford the pure ligand in 90% yield. Its full structural characterization was carried out by nuclear magnetic resonance (NMR) spectroscopy using a Bruker Ultra Shield 500 spectrometer in CDCl_3_ solvent at room temperature.

### Analytical Response Evaluation

To perform colorimetric Cu^2+^ titration experiments, 200 μL of aqueous solutions of Cu^2+^ with concentrations in the range 1–50 μM were added sequentially to 40 μL of the 5 mM BPTP stock solution prepared in ethanol. The pH was adjusted to 7.0 by adding 20 μL of a 0.1 mM NaOH solution, and the sample volume was adjusted to 2 mL. Naked eye colorimetric response was observed upon the addition of Cu^2+^, with a change of the solution’s color from yellow to red. A reaction time of 5 min was allowed after the addition of Cu^2+^ to ensure complete complex formation and stabilization of the colorimetric response before measurement. The absorption spectra were collected with a Genesys 10S UV–vis spectrophotometer from Thermo Scientific using standard quartz cuvette cells of path length 1.0 cm. The absorbance and absorption wavelength values were expressed as the mean and standard deviation (SD) of triplicate assays. The detection limit was calculated using the equation LOD = 3σ/*m*, where σ is the standard deviation of five independent replicates of the blank solution, and *m* is the slope of linear calibration curve obtained by plotting the absorbance intensity at 520 nm versus Cu^2+^ concentration.

For selectivity assays, 5 mM stock solutions of different metal ions including Cu­(NO_3_)_2_·3H_2_O, Na_2_NO_3_, Ca­(NO_3_)_2_·4H_2_O, Mn­(CH_3_CO_2_)_2_, Co­(NO_3_)_2_·6H_2_O, Ni­(NO_3_)_2_·6H_2_O, Zn­(NO_3_)_2_·6H_2_O, Pb­(NO_3_)_2_, AlCl_3_, Cd­(NO_3_)_2_·4H_2_O, K_2_NO_3_, FeSO_4_·7H_2_O, HgCl_2_, CrCl_3_·3H_2_O, and Mg­(NO_3_)_2_·6H_2_O were prepared in distilled water. Test solutions of BPTP were prepared in distilled water by mixing 40 μL of stock BPTP solution with 40 μL of metal ion solutions, followed by dilution to a final volume of 2 mL with water. Competitive experiments were conducted by mixing 10 μL of each metal ion solution with 10 μL of Cu^2+^ solution. The metal ion mixtures were added to the BPTP test solution at pH 7 and adjusted to 2 mL with water. To improve both complexation kinetics and sensitivity, the pH of the test BPTP solution was adjusted in the 4–9 range by titration with 0.1 mM HCl or NaOH. For further experiments, the pH was adjusted to 7.0 by adding 20 μL of a 0.1 mM NaOH solution. The analytical behavior of BPTP toward different cations, cation mixtures and the pH effect was investigated by UV–vis spectroscopy.

### Fluorescence Quenching Experiments of Rhodamine B and Fluorescein

The fluorescence quenching experiments using RhB and fluorescein as fluorophores were conducted. First, assay selectivity probes were carried out by adding 40 μL of metal ion stock solutions and 60 μL of RhB or fluorescein 50 μM stock solutions to the test solution containing the BPTP at pH 7. Then, the RhB system was selected to perform colorimetric and fluorescent titration experiments, where 60 μL of a 50 μM RhB solution and 200 μL of aqueous Cu^2+^ solutions with concentrations ranging from 1 to 50 μM were added sequentially to the BPTP test solutions, adjusted to 2 mL. The detection limit for the fluorescent method was calculated using the equation LOD = 3σ/*m*, where σ is the standard deviation of five independent replicates of the blank solution, and *m* is the slope of the linear calibration curve obtained by plotting the integrated emission intensity versus Cu^2+^ concentration. For the competitive assays, 10 μL of each metal ion solution was mixed with 10 μL of Cu^2+^ solution and added to the BPTP test solution at pH 7, which contained 60 μL of RhB 50 μM. The final volume was adjusted to 2 mL with distilled water. Colorimetric and fluorescent changes were monitored by absorption and emission spectra.

The absorption spectra were collected as described in the previous section and the detection limit for the colorimetric assay using RhB was calculated using the equation LOD = 3σ/*m*, where σ is the standard deviation of five independent replicates of the blank solution, and *m* is the slope of the linear calibration curve obtained by plotting the absorption intensity at 555 nm versus Cu^2+^ concentration.

Emission and average lifetime measurements were carried out using an Edinburgh Instruments FS5 spectrofluorometer with a xenon lamp and a 402.5 nm pulsed laser as excitation source, respectively. Emission spectra were collected using 550 nm as the excitation wavelength, dwell time: 0.1, scan slit: 0.1, offset slit: 0.1 at 20 °C. While decay curves were recorded under pulsed excitation at 20 MHz.

### ESI-MS Characterization

A high-resolution, electrospray ionization-quadrupole-time-of-flight mass spectrometer maXis impact ESI-QTOFMS equipped with Data Analysis 4.1 (Bruker Daltonics) was used for copper complex characterization with sample introduction by direct infusion (3 μL/min). ESI was operated in a positive mode with ion spray voltage 4500 V, dry gas 4 L/min, drying temperature 180 °C and nebulizing gas pressure 0.4 bar. Spectra were acquired using a lock-mass standard *m*/*z* 299.2945 (methyl stearate) in the ion source.

### Computational Analysis

Density functional theory (DFT) calculations of the free ligand BPTP and its BPTP–Cu^2+^ complex were performed using the Gaussian 16 program. Geometries were optimized in the gas phase using the hybrid B3LYP functional.^34^ For dispersion interactions, Grimme’s DFT-D3 dispersion correction was included.^35^ The 6-311G­(d,p) basis set was employed for the ligand atoms, whereas the LANL2DZ effective core potential and basis set were used for the Cu atom. The nature of the stationary points was confirmed by vibrational frequency calculations, and all optimized structures were characterized as local minima by the absence of imaginary frequencies. Solvent effects of water were considered using the SMD solvation model.^36^ Natural bond orbital (NBO) analysis was performed to evaluate donor–acceptor interactions through second-order perturbation energies *E* ^(2)^.^37^ Frontier molecular orbitals (HOMO and LUMO) were analyzed and visualized using Chemcraft 1.8.

### Detection of Cu^2+^ in Tap Water Samples

As proof of concept, the colorimetric chemosensor was used to detect Cu^2+^ in local tap water, using both colorimetric and fluorescence methods. In the colorimetric assay, 200 μL of pure tap water or 5, 10, and 15 μM Cu^2+^ dilutions in the same media were added to BPTP solutions, and the final volume of the samples was adjusted to 2 mL with distilled water. For the fluorescent test, the samples were prepared by adding 200 μL of tap water and 60 μL of RhB stock solution (50 μM) to BPTP solutions. Standard additions of 5, 10, and 15 μM Cu^2+^ solutions prepared in tap water were also analyzed, adjusting the final volume to 2 mL. The absorption and emission spectra were collected. All the experiments were conducted at least in triplicate under the same experimental conditions, and the resulting averages were recorded.

## Results and Discussion

### Synthesis and Structural Characterization of 2-(((6-Bromopyridin-2-yl)­methylene)­amino)-4-(*tert*-butyl)­phenol (BPTP)

The Schiff base was obtained as described in our previous report^33^ by a condensation reaction between 6-bromo-2-pyridinecarboxaldehyde and 2-amino-4-*tert*-butylphenol in ethanol at room temperature, as summarized in Figure 1.

**Figure 1:**
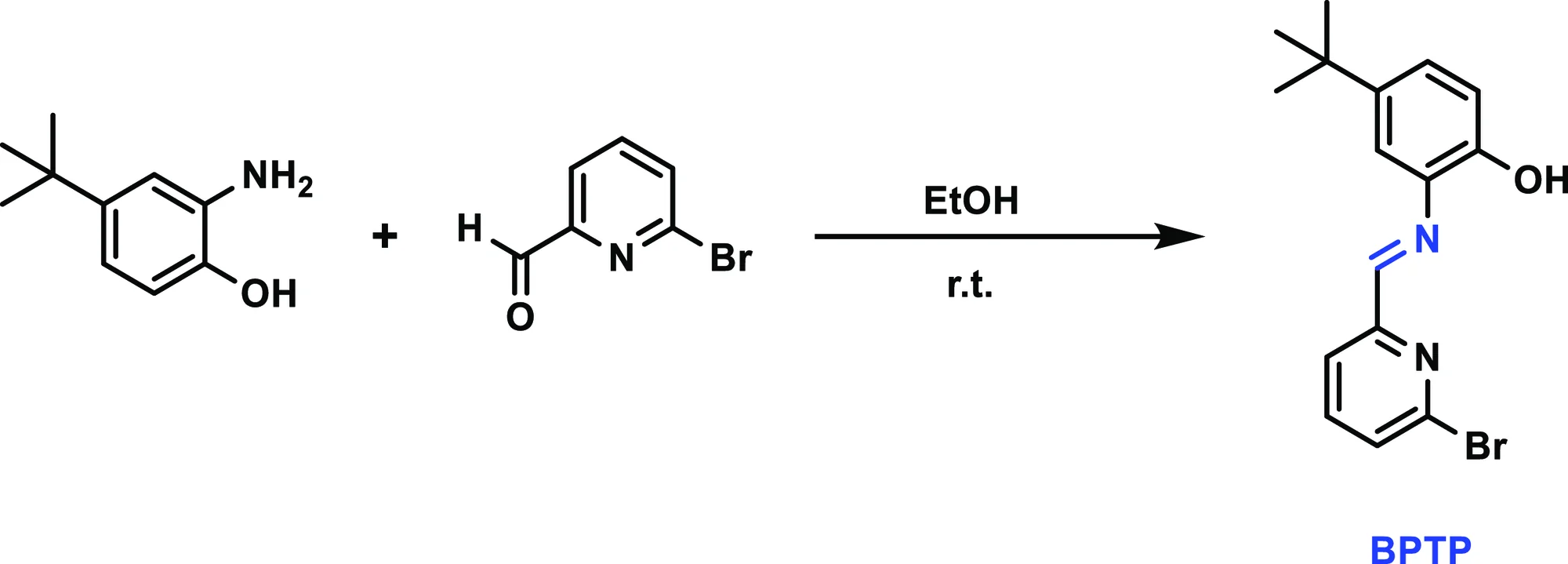
Reaction scheme for the synthesis of 2-(((6-bromopyridin-2-yl)­methylene)­amino)-4-(*tert*-butyl)­phenol (BPTP).

BPTP was obtained as a yellow solid and was fully characterized by 1D and 2D NMR experiments; the RMN spectra are shown in Supporting Information (Figures S1–S7). ^1^H RMN spectrum shows nine expected signals, eight in the aromatic region and one signal at high field assigned to H_h_ methyl protons (Figure S1). The COSY spectrum shows two sets of correlations that distinguish the protons of the pyridin- from the phenol-fragment ring (Figure S4). The first set of signals conforms to a doublet, a triplet, and a doublet at 8.17, 7.68, and 7.56 ppm, assigned to the H_c_, H_b_, and H_a_ protons of the pyridine ring, respectively. Later, the second test of signals includes a doublet of doublets and a doublet at 7.30 and 6.96 ppm, assigned to the H_g_ and H_f_ protons of the phenol ring. Furthermore, the proton H_i_ of the phenol ring appears as a doublet at 7.40 ppm. The protons of the imine- and hydroxyl-group are assigned to singlet signals observed at 8.81 and 7.04 ppm, respectively. Additionally, an intense NOE effect can be observed between methyl protons (H_h_) with H_g_ and H_i_ phenol protons, as well as H_d_ imine proton with H_i_ phenolic proton. Interestingly, a weak NOE effect between H_c_ pyridin-proton with H_d_ imine- and HO-proton is observed, suggesting a rotational process around the C–C single bond on pyridin- and imine-fragments (Figure S8).

### Selective Analytical Response toward Cu^2+^ Determination

To determine the optimal conditions for the selective recognition of Cu^2+^, the analytical response of BPTP was systematically evaluated in the presence of various metal ions across a broad pH range. At neutral pH, BPTP exhibits colorimetric selectivity for Cu^2+^, characterized by a significant color change from yellow to red that is visible to the naked eye. This behavior was not observed for the other ions tested (Figure 2a). For a complementary and systematic visualization of the colorimetric selectivity for Cu^2+^ at pH 7, the CIELAB coordinates were obtained. The CIELAB parameters were calculated from the absorption spectra, considering the standard D65 illuminant and a 2° standard observer, enabling a quantitative assessment of the perceived color changes upon metal ion coordination. The BPTP ligand exhibits CIELAB coordinates located within the yellow–green region (a* < 0, b* > 0), consistent with its characteristic absorption in the violet–blue region of the visible spectrum. Upon interaction with most of the evaluated metal ions, the resulting coordinates remain relatively close to those of the free ligand, indicating only minor chromatic variations. In contrast, the presence of Cu^2+^ induces a pronounced shift toward positive a* values, accompanied by a significant decrease in b*. This displacement reflects a clear transition from a yellow–green hue to a reddish tone with reduced yellow contribution. Such a marked chromatic response correlates well with the emergence of a broad absorption band in the visible region, attributed to the formation of the corresponding coordination complex. These results highlight that Cu^2+^ generates the most distinguishable colorimetric response among the tested analytes. It is worth noting that Pb^2+^ also produces a measurable perturbation in the CIELAB coordinates, which may represent a potential source of interference in purely colorimetric detection. However, this limitation is effectively addressed by measuring the absorption spectra (Figure 2b). Although Pb^2+^ triggers a marked decrease in the intensity of the yellow color, the absorption profiles of the Pb^2+^ and Cu^2+^ complexes are well-resolved, ensuring the selective detection of copper without spectral overlapping. This selective behavior remains consistent under basic conditions (pH 8 and 9, Figure S9). In contrast, at pH 6, the absorption bands of the Pb^2+^ and Cu^2+^ complexes overlap, hindering the selective quantification of the copper ion (Figure S10). A further decrease in pH to 5 introduces additional interference from Cd^2+^ and Hg^2+^, which exhibit significant spectral overlaps with the copper complex (Figure S11). Under these conditions (pH 5), these cations can compete with protons for coordination with the imine nitrogen, generating a lack of spectral resolution that avoids selective detection. Finally, at pH 4, both the metal-free BPTP and the metal-containing solutions appear colorless, including the copper complex (Figure S12). At this acidic threshold, the protonation of the imine nitrogen is favored, promoting the hydrolysis of the C=N bond and the subsequent regeneration of the amine and aldehyde precursors. This hydrolytic cleavage disrupts the chromophore’s π-conjugation, resulting in a decrease in absorption in the visible region and a shift to the high-energy ultraviolet region. According to these results, the selective and robust detection of copper is restricted to pH values equal to or greater than 7.

**Figure 2:**
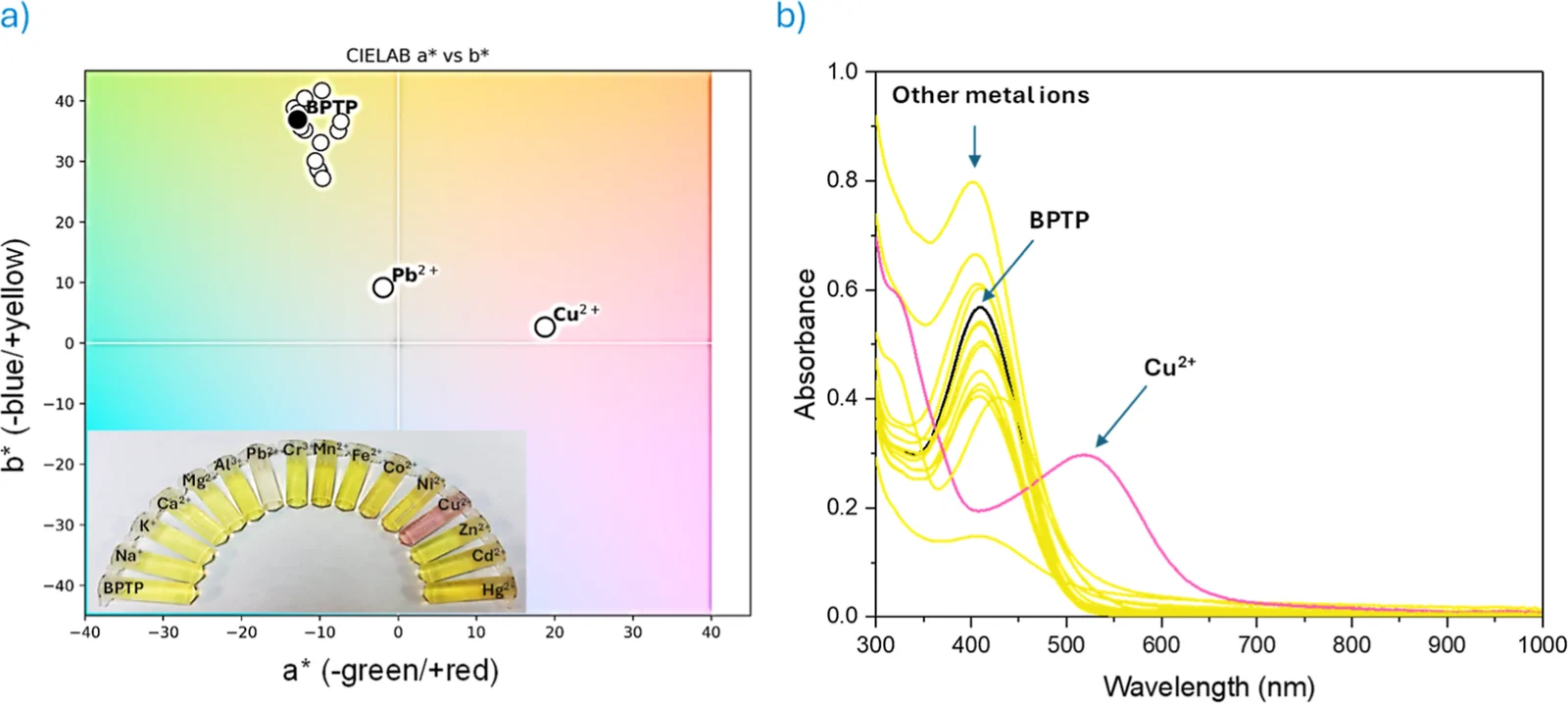
Analytical response of BPTP (100 μM) in the presence of different metal ions (100 μM) in water at pH 7. (a) CIELAB coordinates and (b) absorption spectra.

To evaluate the effect of potentially competing metal ions on Cu^2+^ detection, competitive binding experiments were carried out in the presence of environmentally and biologically relevant cations (Figure S13). Changes in the absorbance at 520 nm, corresponding to the BPTP–Cu complex, were monitored to assess the interference effects of competing cations (Figure S13a). Most of the tested ions, including alkali metal ions (Na^+^ and K^+^), alkaline earth metal ions (Ca^2+^ and Mg^2+^), Al^3+^, and several transition metal ions, produced negligible changes in the absorbance intensity. In contrast, the simultaneous presence of Cr^3+^ or Hg^2+^ led to a noticeable decrease in absorbance at 520 nm, indicating partial interference with the copper-sensing response. Meanwhile, Co^2+^ and Ni^2+^ induced a hypsochromic shift together with an increase in the absorption intensity. These effects are more clearly illustrated in Figure S13b. These results demonstrate that BPTP exhibits good selectivity toward Cu^2+^ in the presence of the most common metal ions. Although Co^2+^, Ni^2+^, Cr^3+^, and Hg^2+^ produce detectable spectral changes, the combined absorption and fluorescence responses remain sufficiently distinct to allow reliable identification of Cu^2+^ under competitive conditions.

### Colorimetric Titration of BPTP (100 μM) with Cu^2+^ in Water at pH 7

To evaluate the binding properties of the BPTP with Cu^2+^, UV–vis titrations were performed by adding Cu^2+^ to the BPTP at pH 7 in water (Figure 3). Upon addition of Cu^2+^ to a solution of BPTP, the absorbance peak at 409 nm gradually decreased, and a new peak appeared at 420 nm, resulting in a color change from yellow to red (Figure 3a). The chemosensor saturation occurs at a BPTP–Cu^2+^ ratio of 2:1, suggesting that ML_2_-type complexes are formed.^23^ High-resolution ESI-MS analysis confirmed this stoichiometry, showing a monoisotopic peak at 726.027 *m*/*z*, which corresponds to the protonated molecular ion of the complex [Cu­(BPTP)_2_]^+^. Furthermore, the characteristic isotopic distribution pattern of copper was clearly resolved and perfectly matched the theoretical simulation (Figure S14). The detection limit was calculated to be 2.1 μM, considering only the linear region (5–40 μM) of the calibration curve using the relation LOD = 3σ/*s* (Figure 3b), where σ is the standard deviation of the blank and *s* is the slope of the calibration curve. This concentration interval was selected because it exhibited a statistically significant linear relationship between signal and analyte concentration.

**Figure 3:**
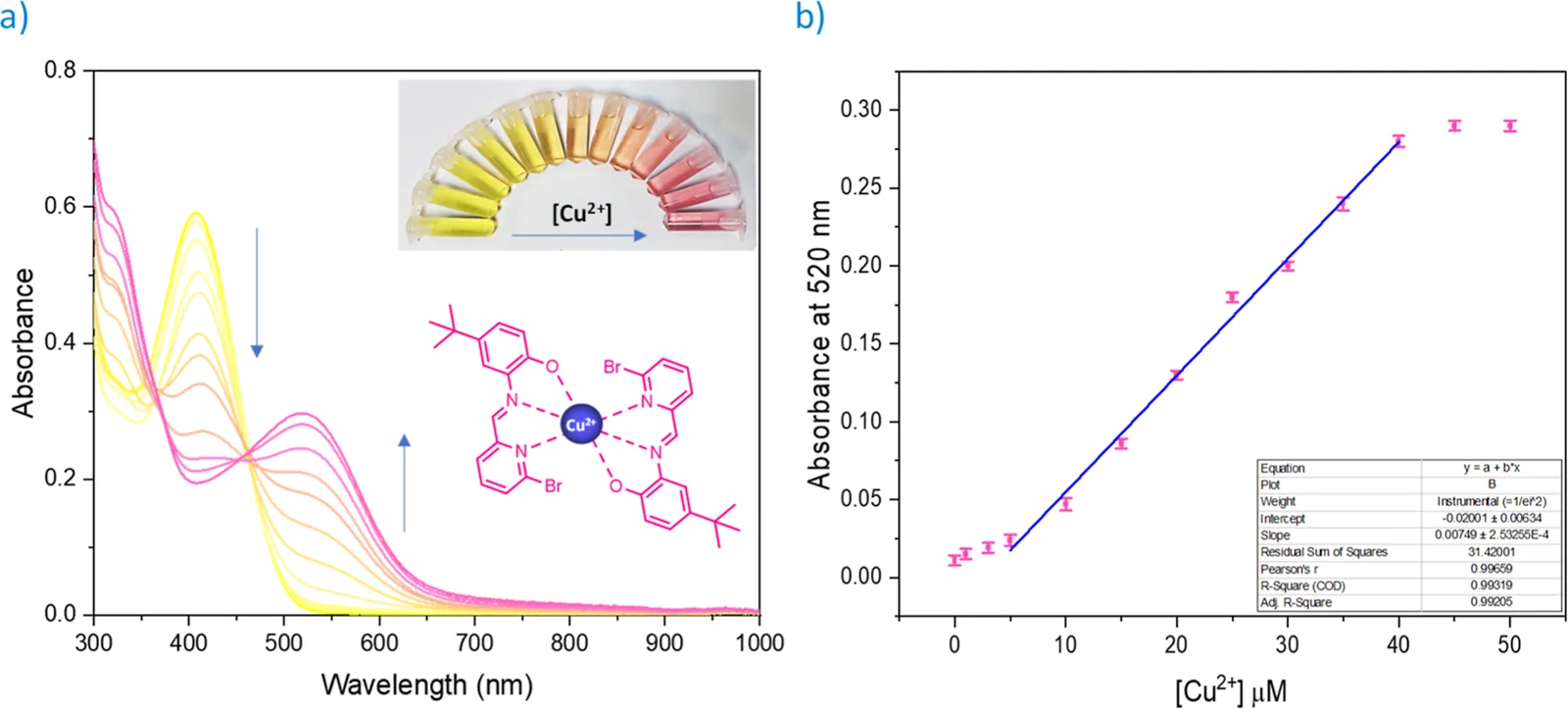
(a) Absorption spectra of BPTP (100 μM) upon addition of Cu^2+^ (1–50 μM) in water at pH 7; the direction of the arrows indicates an increase in the Cu^2+^ concentration and (b) the corresponding calibration curve.

### Colorimetric–Fluorescent Dual-Mode Probe Cu^2+^ Chemosensor

To develop a dual colorimetric–fluorescent probe, the photoluminescent properties of RhB and fluorescein fluorophores were evaluated in the presence of the BPTP and the BPTP–Cu^2+^ complex under 550 nm visible light excitation (Figure 4a,b). Those molecules were selected because of the overlap between the absorption band of the copper complex and the emission bands of fluorescein, and RhB creates an emitter–absorber system in which the RhB quenching by IFE can be exploited for copper sensing through variations in fluorescence (Figure 4c). Indeed, the absorbance of free BPTP does not overlap with the emission of fluorescein or RhB; however, the bathochromic shift of the absorption band resulting from the formation of the BPTP–Cu^2+^ complex induces spectral coupling, enabling an IFE effect and, consequently, a correlation of the fluorophore emission intensity with the Cu^2+^ concentration.

**Figure 4:**
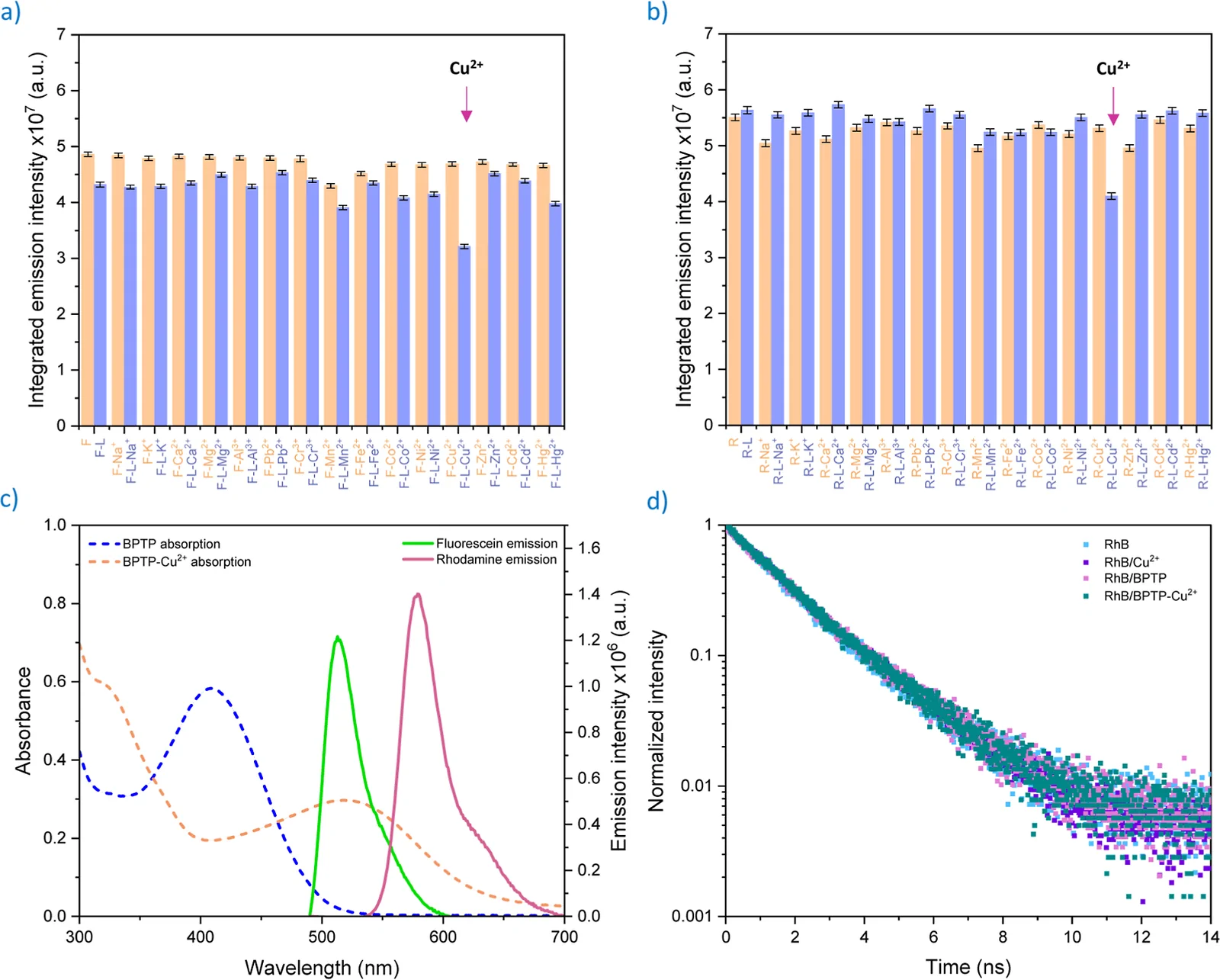
Integrated emission intensity of (a) fluorescein and (b) RhB, in the presence of metal ions (orange bars) and BPTP–metal complexes (blue bars). F: fluorescein, FL: fluorescein + BPTP ligand, R: RhB, and RL: RhB + BPTP ligand. (c) Absorption bands of BPTP (100 μM) and the BPTP–Cu^2+^ complex (2:1 ratio) overlapping with the emission bands of RhB (2.5 μM) and fluorescein (2.5 μM), λ_ex_ = 550 nm in water at pH 7. (d) Decay time spectra of RhB, RhB/Cu^2+^, RhB/BPTP, and RhB/BPTP–Cu^2+^.

The UV–vis spectra of RhB samples in the presence of metal ions (Figure S15a) show no colorimetric changes with respect to pure RhB. Similar behavior is observed for RhB in the presence of the BPTP–Cu^2+^ complexes (Figure S15b), where most retain the orange color resulting from the RhB + BPTP mixture, except for lead and copper, which exhibit red coloration. Under these conditions and by naked-eye observation, lead may act as a colorimetric interferent. However, the signals arising from the presence of copper are more intense, and the two can be distinguished by monitoring the absorption band at 400 nm, which appears in the presence of lead but is absent in the presence of Cu^2+^.

On the other hand, the emission of fluorescein and RhB in the presence of different metal ions and their corresponding complexes was also investigated (Figure S16). For the fluorescein-based system, while the emission intensity remained relatively stable in the presence of free metal ions, a generalized decrease in fluorescence was observed with the complexes. Notably, the copper complex induced the most significant quenching effect, consistent with previous observations (Figure 4a). Although this analytical response may be distinguishable, the significant attenuation of fluorescein luminescence in the presence of different cations could lead to false positives, hindering its practical implementation. In contrast, RhB exhibits a lower variation in the presence of the metal ions other than Cu^2+^ and shows a selective fluorescence attenuation response toward the copper complex (Figure 4b). Thereby, the RhB/BPTP system was selected for further experiments.

To maximize analytical performance in the RhB-system chemosensor, a systematic study was conducted to determine the optimal RhB concentration. Concentrations ranging from 0.5 to 2.5 μM were evaluated to assess their effect on the quenching efficiency of photoluminescence upon Cu^2+^ addition (Figure S17). The relative emission intensity was determined by calculating the ratio of the integrated area of the RhB/BPTP–Cu^2+^ system to that of free RhB. This analysis identified a higher sensitivity range between 1.5 and 2.5 μM, observing attenuations between 22.2 and 25.8%. Considering that there were no significant differences between these treatments and that the required RhB concentration can be reduced, 1.5 μM was established as the optimal concentration to minimize self-absorption phenomena, thereby ensuring a more reliable and accurate analytical response.

To elucidate the operating mechanism of the chemosensor, fluorescence lifetime measurements were analyzed using a single-exponential decay model. The fitted lifetimes were 1.67 ns for free RhB, 1.69 ns for RhB/Cu^2+^, 1.69 ns for the RhB/BPTP system, and 1.65 ns for the integrated RhB/BPTP–Cu^2+^ system (Figure 4d). The negligible variation in the fluorescence lifetime indicates that the excited-state deactivation dynamics remain essentially unchanged upon the addition of BPTP and Cu^2+^, suggesting that the fluorescence attenuation does not arise from dynamic quenching. The invariance of the excited-state lifetime indicates that the selective quenching of RhB proceeds by an inner filter effect (IFE) mechanism and not a resonance energy transfer (FRET) (Figure 5). To verify the IFE mechanism, the IFE was quantitatively corrected according to the Lakowicz equation^38^

$$F_{corr}=F_{obs}\;antilog\left(\frac{A_{exc}+A_{em}}{2}\right)$$

where *F*_obs_ is the measured fluorescence *A*_exc_ and *A*_em_ are the absorbance at the excitation (550 nm) and emission (580 nm) wavelengths, respectively. Using the absorbance values summarized in Table S1, the maximum fluorescence intensity of the spectra shown in Figure 6a was corrected for both primary and secondary inner filter effects. To evaluate the contribution of IFE to the fluorescence attenuation, the normalized fluorescence intensity (*F*/*F*_0_) at 580 nm was calculated before and after correction. The results are presented in Figure S18. As expected, the fluorescence intensity decreased progressively with increasing Cu^2+^ concentration due to the growth of the absorption band associated with the BPTP–Cu^2+^ complex (500 to 600 nm). This band significantly overlaps with both the excitation and emission regions of RhB, providing the optical basis for IFE-mediated fluorescence modulation. After applying the Lakowicz correction, a substantial recovery of the fluorescence signal was observed. At 50 μM Cu^2+^, the uncorrected fluorescence intensity decreased to 57.5% of its initial value, whereas the corrected fluorescence retained 82.8% of the original intensity. This significant recovery demonstrates that the fluorescence attenuation is predominantly governed by the inner filter effect generated by the BPTP–Cu^2+^ absorbing species. It should be noted that the classical Lakowicz correction is an approximation that assumes moderate absorbance values and a homogeneous optical path. Previous studies have shown that its accuracy decreases as the absorbance increases, particularly when the total absorbance approaches values near 0.3.^38–40^ In the present system, the combined absorbance at the excitation and emission wavelengths approaches this range at concentrations above 15 μM of Cu^2+^ (Table S1), which may contribute to the residual decrease observed after correction. The absorption spectra of Figure 6c reveal the progressive growth of the BPTP–Cu^2+^ absorption band without the appearance of new spectral features attributable to RhB complexation. Likewise, the emission profile of RhB (Figure 6a) remains unchanged throughout the titration, indicating that its excited-state electronic structure is not significantly perturbed. Furthermore, the fluorescence lifetime remains essentially constant after Cu^2+^ addition. Taken together, these observations rule out dynamic quenching and make a FRET-type mechanism highly unlikely, supporting IFE as the dominant fluorescence modulation process in the RhB/BPTP–Cu^2+^ system. Although minor secondary contributions cannot be completely excluded, the combined evidence from spectral overlaps, fluorescence lifetime measurements, and quantitative IFE correction strongly indicates that the observed fluorescence response is mainly controlled by IFE.

**Figure 5:**
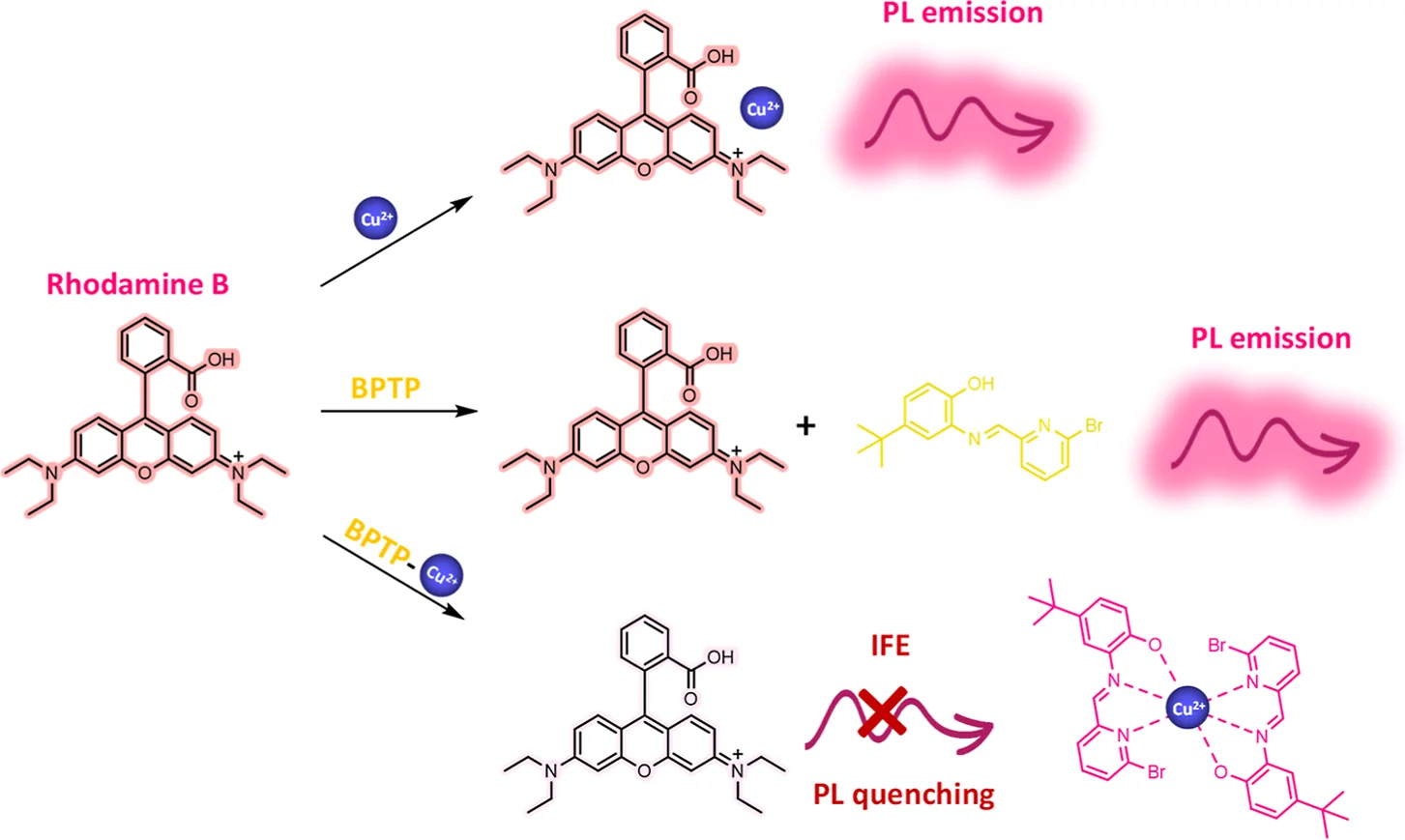
Illustration of the quenching mechanism induced by the inner filter effect (IFE).

**Figure 6:**
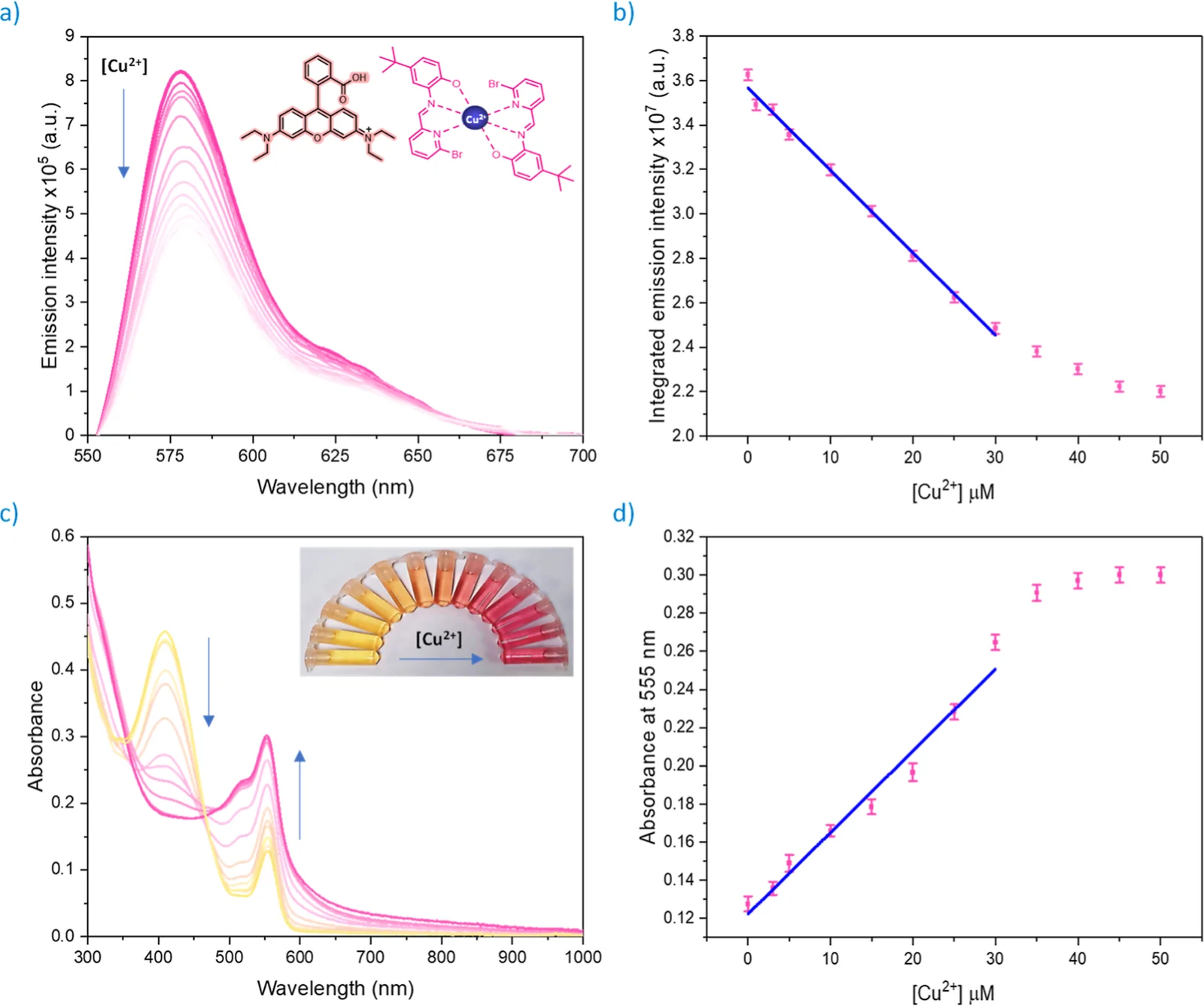
(a) Emission spectra of RhB/BPTP upon addition of Cu^2+^ (λ_ex_ = 550 nm) and (b) the corresponding calibration curve. (c) Absorption spectra of RhB/BPTP upon addition of Cu^2+^ and (d) the corresponding calibration curve. [RhB] = 1.5 μM, [BPTP] = 100 μM, in water at pH 7; the direction of the arrows indicates an increase in the Cu^2+^ concentration.

To explore the analytical performance of the dual-mode platform, fluorescence and colorimetric titration analyses were conducted using a RhB (1.5 μM)/BPTP (100 μM) system by sequentially adding different concentrations of Cu^2+^ in the range of 0 to 50 μM (Figure 6). As shown in Figure 6a, a gradual decrease in the emission intensity was observed upon the addition of Cu^2+^, reaching a minimum at the molar ratio of 2:1 of RhB/BPTP–Cu^2+^ ([Cu^2+^] = 50 μM). In the linear range from 0 to 30 μM (Figure 6b), the detection limit was calculated to be 1.9 μM (Table S2).

Interference experiments revealed complementary behavior observed in colorimetric assays. Most alkali, alkaline earth, and transition-metal ions caused only minor variations in emission intensity, indicating limited interference with the fluorescence response of the Cu^2+^ complex. However, Co^2+^, Ni^2+^, Cr^3+^, and Hg^2+^ produced a measurable enhancement of the emission signal relative to that of the Cu^2+^ complex, as expected (Figure S19a,b).

On the other hand, in the colorimetric titration, the absorption band at 409 nm decreases upon the addition of Cu^2+^, while the absorption band at 555 nm increases (Figure 6c). In both cases, the chemosensor saturates at a 1:2 ratio ([Cu^2+^] = 50 μM), as expected given the stoichiometry of the BPTP–Cu^2+^ complex (Figure 6d). The detection limit for this system was calculated to be 2.9 μM based on the data (Table S3) in the linear range from 0 to 30 μM, considering the 555 nm band. These values are comparable to and competitive with those previously reported in the literature, as shown in Table S4. Notably, the chemosensor developed in this work offers the advantage of being easily synthesized in large quantities at room temperature and it does not require a purification process. Furthermore, its mechanism is based on a dual-mode approach (colorimetric and luminescent) that offers complementary detection pathways for Cu^2+^ recognition. Additionally, its ability to operate in aqueous environments significantly broadens its range of potential applications.

### Density Functional Theory (DFT) and Natural Bond Orbital (NBO) Studies

Theoretical studies based on density functional theory (DFT) were performed to gain insight into the binding mechanism of BPTP toward Cu^2+^ ion. The optimized geometries of BPTP and BPTP–Cu^2+^ complex are shown in Figure S20. The BPTP ligand acts as a tridentate chelator, coordinating to the Cu^2+^ center through the hydroxyl oxygen atom, the imine nitrogen atom, and the pyridine nitrogen atom. This coordination mode leads to the formation of a homoleptic BPTP–Cu^2+^ complex showing a distorted octahedral geometry around the metal center.

The natural bond orbital (NBO) second-order perturbation analysis confirms the multidentate coordination of BPTP to the Cu^2+^ ion. Significant interactions between the lone-pair electrons of the oxygen and nitrogen donor atoms and the vacant orbitals of Cu^2+^ were observed, demonstrating strong ligand-to-metal electron donation.

Frontier molecular orbital (FMO) analysis provided further insight into the optical sensing mechanism and the experimentally observed colorimetric response upon Cu^2+^ complexation. For the free BPTP ligand, both the highest occupied molecular orbital (HOMO) and the lowest unoccupied molecular orbital (LUMO) are delocalized over the π-conjugated aromatic framework. Upon coordination with Cu^2+^, a significant redistribution of the frontier orbitals takes place. The HOMO remains mainly localized on the π-conjugated ligand framework, whereas the LUMO becomes mainly centered on the copper ion. This electronic reorganization is accompanied by a reduction in the HOMO–LUMO energy gap, from 4.06 eV for the free ligand to 2.63 eV for the BPTP–Cu^2+^ complex in water. This decrease in the energy gap is consistent with the experimentally observed bathochromic shift and the pronounced color change associated with complex formation (Figure 7).

**Figure 7:**
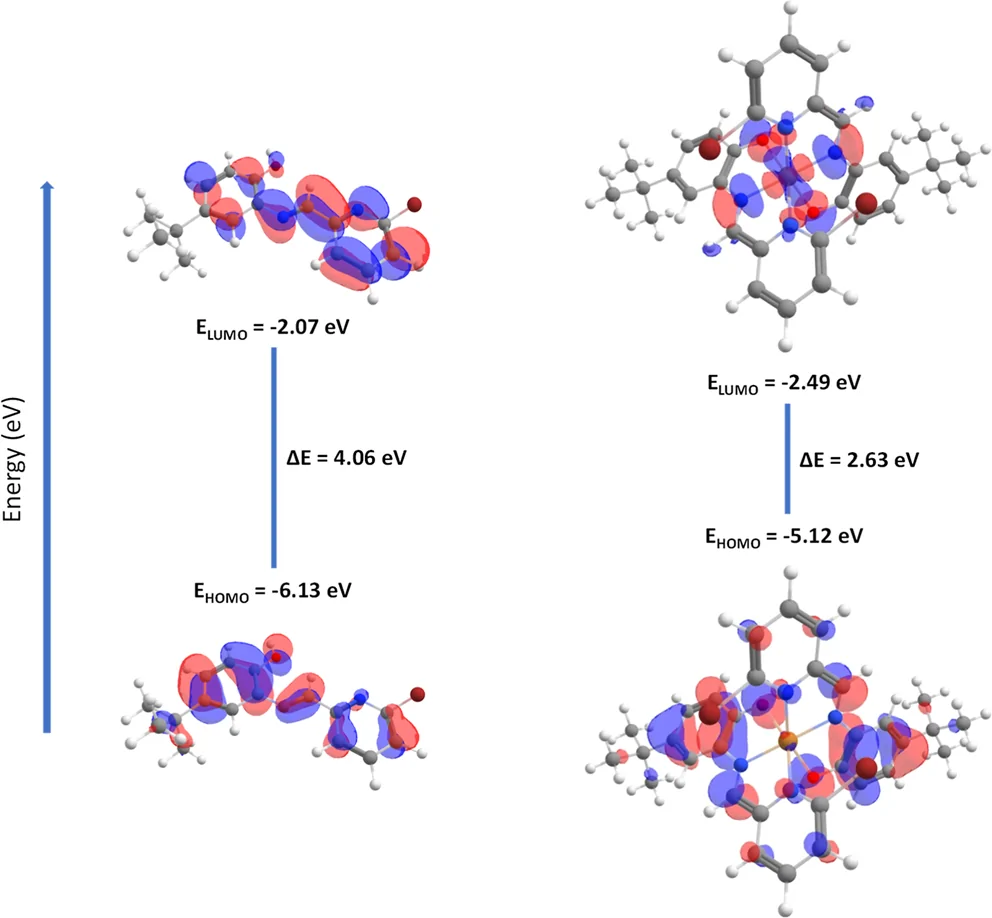
Schematic representation of HOMO and LUMO levels of the BPTP and BPTP–Cu^2+^ complex.

The calculated HOMO and LUMO energies, energy gaps (Δ*E*), selected bond lengths and bond angles, as well as the NBO second-order perturbation energies (*E* ^(2)^) for BPTP and BPTP–Cu^2+^ complex in both gas and water phases are summarized in Table 1.

**Table 1:** Energy Parameters, Second-Order Perturbation Theory Analysis of the Fock Matrix in the NBO and Selected Bond Lengths of BPTP and BPTP–Cu^2+^ Complex

system	phase	*E*_HOMO_ (eV)	*E*_LUMO_ (eV)	Δ*E* (eV)	NBO_donor→acceptor_	*E* ^(2)^ (kcal/mol)	bond length (Å)
BPTP	gas	–6.05	–2.09	3.96	---	---	---
BPTP	water	–6.13	–2.07	4.06	---	---	---
BPTP–Cu^2+^	gas	–4.74	–2.27	2.47	LP(O_hydroxyl_) → Cu	64.7	1.97
					LP(N_imine_) → Cu	39.4	2.01
					LP(N_pyridine_) → Cu	38.5	2.28
BPTP–Cu^2+^	water	–5.12	–2.49	2.63	LP(O_hydroxyl_) → Cu	49.7	1.95
					LP(N_imine_) → Cu	33.4	2.00
					LP(N_pyridine_) → Cu	20.5	2.42

### Cu^2+^ Detection in Tap Water

To validate the practical applicability of the copper chemosensor, the concentration of Cu^2+^ in local tap water was quantified using both colorimetric and fluorescence methods. For the colorimetric assay, BPTP was evaluated in the absence of RhB to isolate its specific absorption response. The experimental procedure also involved the standard addition approach, consisting of spiking tap water samples with known concentrations of Cu^2+^, specifically 0, 5, 10, and 15 μM. That experiment allowed to evaluate potential matrix effects. To ensure high analytical accuracy, all measurements were conducted within the previously established linear range. Figure 8 shows the absorbance and emission profiles for the BPTP chemosensor in the presence of pure and spiked tap water samples, and the comparison with the response observed upon addition of 0, 5, 10, and 15 μM Cu^2+^ solutions prepared in distilled water. In the colorimetric analysis, a measurable increase in absorbance at 520 nm was observed upon addition of the tap water samples compared to samples prepared with Cu^2+^ dissolved in distilled water (Figure 8a). Conversely, the fluorometric method revealed a consistent decrease in emission intensity relative to the samples prepared in distilled water, attributed to the fluorescence quenching of RhB (Figure 8b). These spectral variations are ascribed to the intrinsic copper content present in the tap water matrix and allowed the calculation of its concentration.

**Figure 8:**
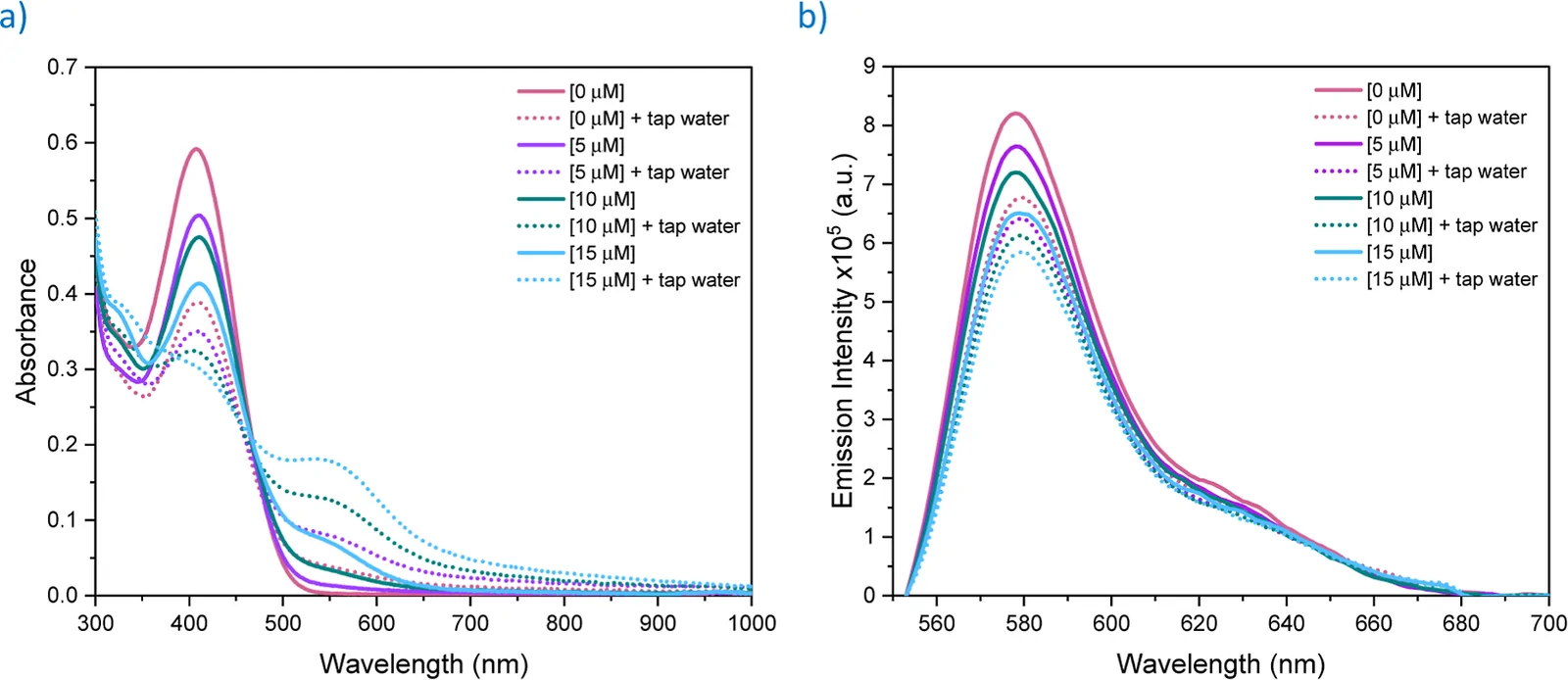
Effect of adding an aliquot of tap water to a known concentration of Cu^2+^ (5–15 μM), (a) on the absorbance intensity at 520 nm and (b) on the emission intensity.

The results summarized in Table 2 showed an average Cu^2+^ concentration in tap water of 8.95 ± 0.93 μM (0.57 ppm) through the colorimetric method and 12.38 ± 1.23 μM (0.78 ppm) using the fluorometric approach, considering pure tap water and the three standard additions. The recovery values for the colorimetric method ranged from 101.4 to 105.9%, whereas those obtained by fluorescence varied from 91.1 to 96.2%, demonstrating satisfactory precision and good repeatability. These results indicate that the proposed sensor is suitable for the determination of Cu^2+^ in tap water, with the colorimetric mode providing higher analytical accuracy under the evaluated conditions. Furthermore, the results obtained using both the colorimetric and fluorescent methods remain well within the safety limits established by the World Health Organization (WHO), which sets the permissible limit for drinking water at 1.3 ppm.^41,42^

**Table 2:** Determination of Cu^2+^ in Tap Water (*n* = 4)

	spiked (μM)	found (μM) (spiked + intrinsic) ± SD	recovery (%)	RDS (%)
Colorimetric Detection				
sample 1	0	8.95 ± 0.93	---	10.4
sample 2	5	14.15 ± 0.87	101.4	6.1
sample 3	10	20.02 ± 1.21	105.6	6.0
sample 4	15	25.37 ± 1.34	105.9	5.3
Fluorescent Detection				
sample 1	0	12.38 ± 1.23	---	9.9
sample 2	5	16.16 ± 0.74	92.9	4.6
sample 3	10	20.39 ± 0.62	91.1	3.0
sample 4	15	26.36 ± 1.02	96.2	3.8

## Conclusions

In this study, a dual-mode Cu^2+^ sensing platform was developed by integrating the Schiff base ligand BPTP with rhodamine B (RhB), enabling complementary colorimetric and fluorescence readouts in aqueous media. The incorporation of a *tert*-butyl substituent into the ligand structure enhanced solubility and facilitated direct operation in environmentally relevant water samples. The sensing response is strongly pH-dependent, with optimal performance achieved at pH ≥ 7.0. A well-defined 2:1 BPTP–Cu^2+^ coordination stoichiometry was consistently validated by UV–vis titration, ESI-MS, and DFT calculations, confirming the robustness of the molecular recognition mechanism. The RhB component introduces an independent fluorescence transduction pathway governed by an inner filter effect (IFE), arising from spectral overlap between RhB and the Cu^2+^-induced absorption band of BPTP. Steady-state and time-resolved fluorescence measurements show invariant lifetimes across all systems, conclusively ruling out dynamic quenching and supporting IFE as the dominant mechanism. This is further corroborated by Lakowicz-based corrections, which demonstrate substantial recovery of fluorescence intensity at high Cu^2+^ concentrations, confirming the optical filtering (IFE) nature of the response. The fluorescence (BPTP + RhB) mode delivers superior selectivity and sensitivity (LOD = 1.9 μM), compared to the colorimetric (BPTP) response (LOD = 2.1 μM). Decay-time measurements further confirm the absence of excited-state perturbation upon Cu^2+^ addition. Finally, the dual platform was successfully applied to tap water analysis, yielding a Cu^2+^ concentration of 12.38 ± 1.23 μM (0.78 ppm), within WHO regulatory limits. Overall, the RhB/BPTP system provides a robust, selective, and chemically modular dual-mode sensing strategy, offering a reliable real-time copper monitoring platform for environmental applications.

## Supplementary Material



## Notes

Figures S1–S3 (^1^H, ^13^C­{^1^H}, DEPT NMR), Figures S4–S7 (2D NMR: COSY, NOESY, HSQC, HMBC) and Figure S8 (rotameric equilibrium) of BPTP in CDCl_3_ at room temperature; Figures S9–S12 (pH-dependent metal-ion response); Figures S13 and S19 (competitive assays); Figure S14 (ESI-MS of BPTP–Cu complex); Figures S15–S18 (UV–vis, photoluminescence, IFE correction, and fluorescence response with RhB/fluorescein); Figure S20 (DFT-optimized structures); and Tables S1–S4 (absorbance data, calibration curves, and comparison of Cu^2+^ chemosensors) (PDF)
