# Supplementary material for: Dual-Mode Colorimetric and Fluorescent Detection of Cu2+ Based on a Two-Component Sensing Platform Integrating an Imine Derivative and Rhodamine B
Source: ACS Omega. 2026 Sep 23;11(39):60053–63. doi: 10.1021/acsomega.6c07479 (PMC13647929; doi:10.1021/acsomega.6c07479)
Supplement: Supplementary file 1 [file ao6c07479_si_001.pdf]

Supplementary Information

Dual-Mode Colorimetric and Fluorescent Detection of Cu^{2+} Based on a Two-Component Sensing Platform Integrating an Imine Derivative and Rhodamine B

Rosa M. Sevillano-Arredondo[1], Jorge Molina-González[1], Marlene Vargas-Zamarripa[2], Oracio Serrano[2]*, Gonzalo Ramírez-García[1]*

[1] Universidad Nacional Autónoma de México. Centro de Física Aplicada y Tecnología Avanzada. 3001, Boulevard Juriquilla, Querétaro, 76000, México.

[2] Universidad de Guanajuato, División de Ciencias Naturales y Exactas. Departamento de Química, Cerro de la Venada s/n, Guanajuato, 36050, México.

***Corresponding authors' email:** oraciosinh@ugto.mx ; gonzalo.ramirez@fata.unam.mx

NMR Spectroscopy data

2-(((6-bromopyridin-2-yl)methylene)amino)-4-(tert-butyl)phenol (BPTP). Yield: 90 %. ^{1}H NMR ($CDCl_3$, 500 MHz): δ = 8.81 (s, 1H), 8.17 (d, 1H, $^3J_{HH}$= 7.68 Hz), 7.68 (t, 1H, $^3J_{HH}$ = 7.73 Hz), 7.56 (d, 1H, $^3J_{HH}$= 7.73 Hz), 7.40 (d, 1H, $^3J_{HH}$= 2.21 Hz), 7.30 (dd, 1H, $^3J_{HH}$= 8.53 Hz, $^4J_{HH}$= 2.21 Hz), 7.04 (s, 1H), 6.96 (d, 1H, $^3J_{HH}$= 8.53 Hz), 1.31 (s, 9H) ppm. $^{13}C\{^1H\}$ NMR ($CDCl_3$, 125 MHz): 155.73, 154.96, 150.70, 143.53, 142.01, 139.09, 133.35, 129.65, 127.92, 119.95, 115.08, 113.05, 34.55, 31.62 ppm.

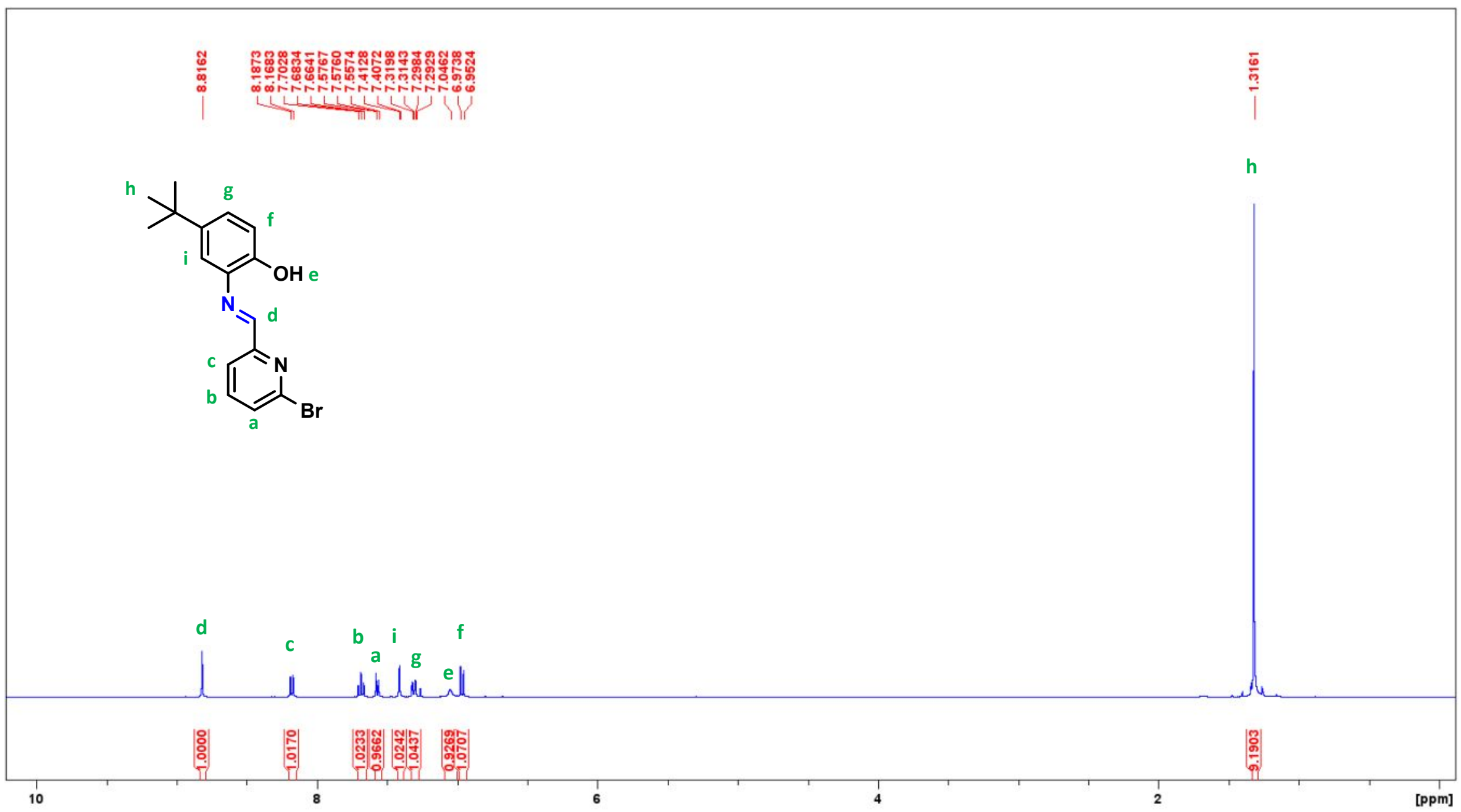

Figure S1. ^{1}H NMR spectrum of BPTP in $CDCl_3$.

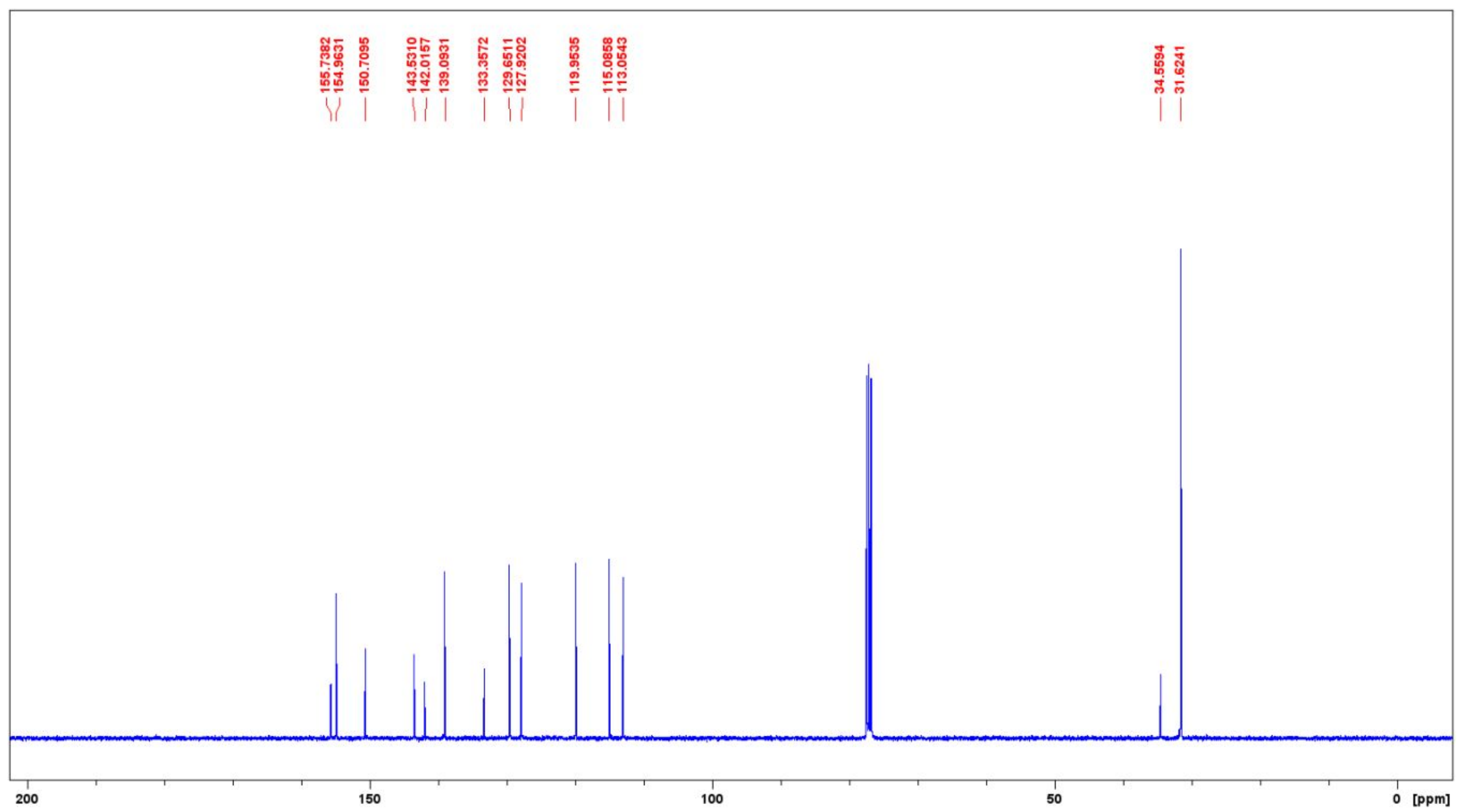

Figure S2. $^{13}C\{^{1}H\}$ NMR spectrum of BPTP in $CDCl_3$.

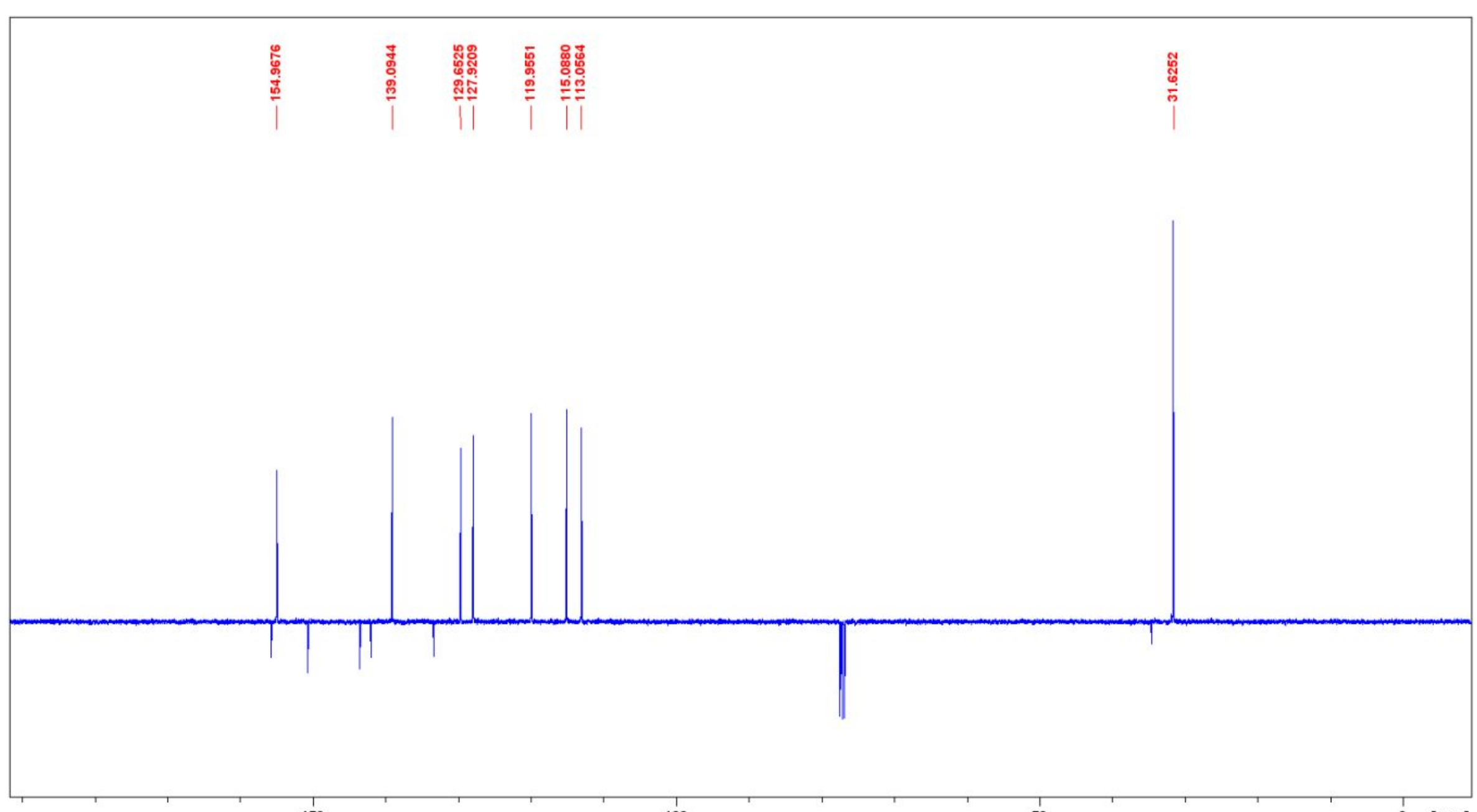

Figure S3. DEPT NMR spectrum of BPTP in CDCl3.

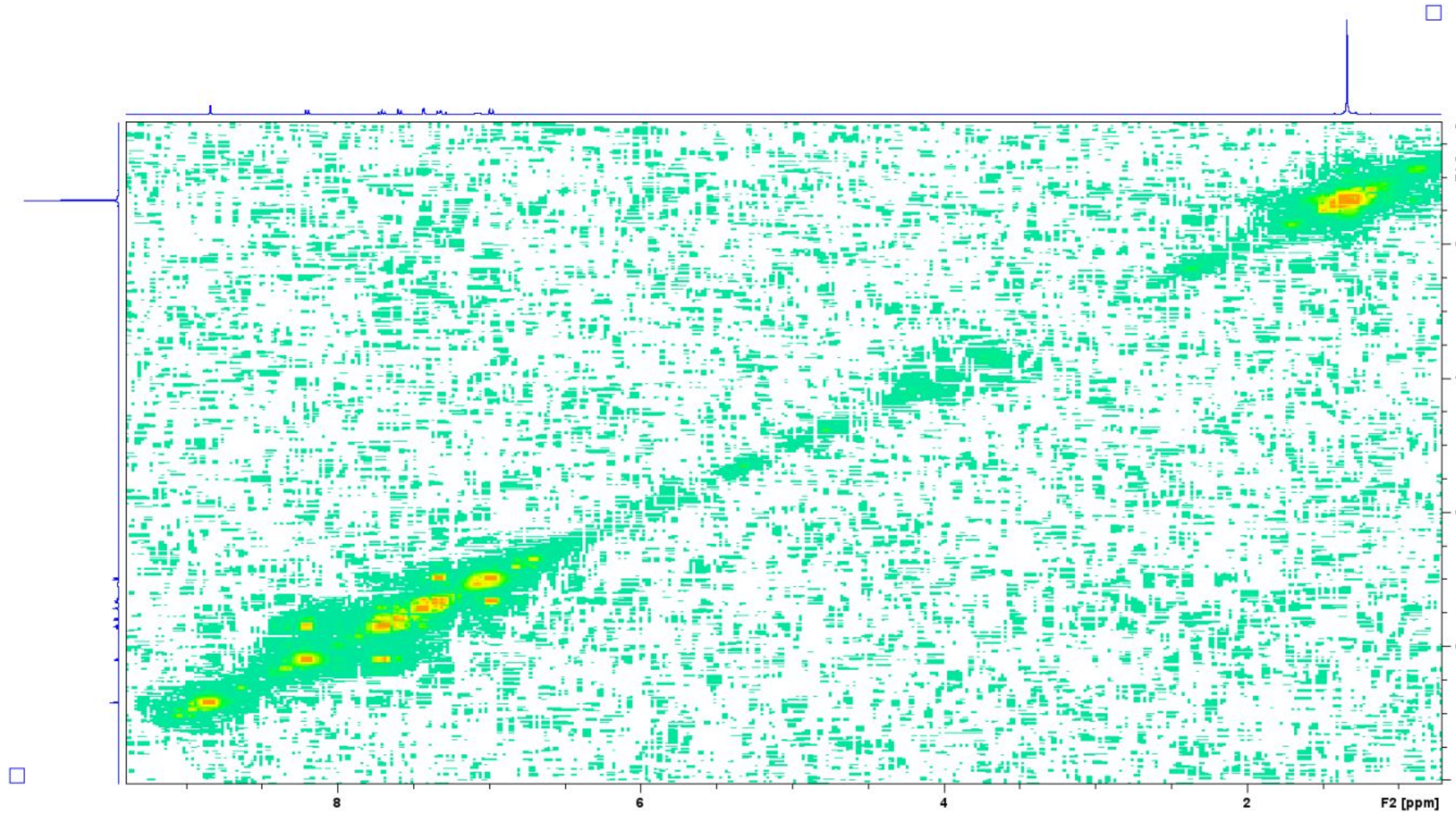

Figure S4. COSY spectrum of BPTP in $CDCl_3$ at room temperature.

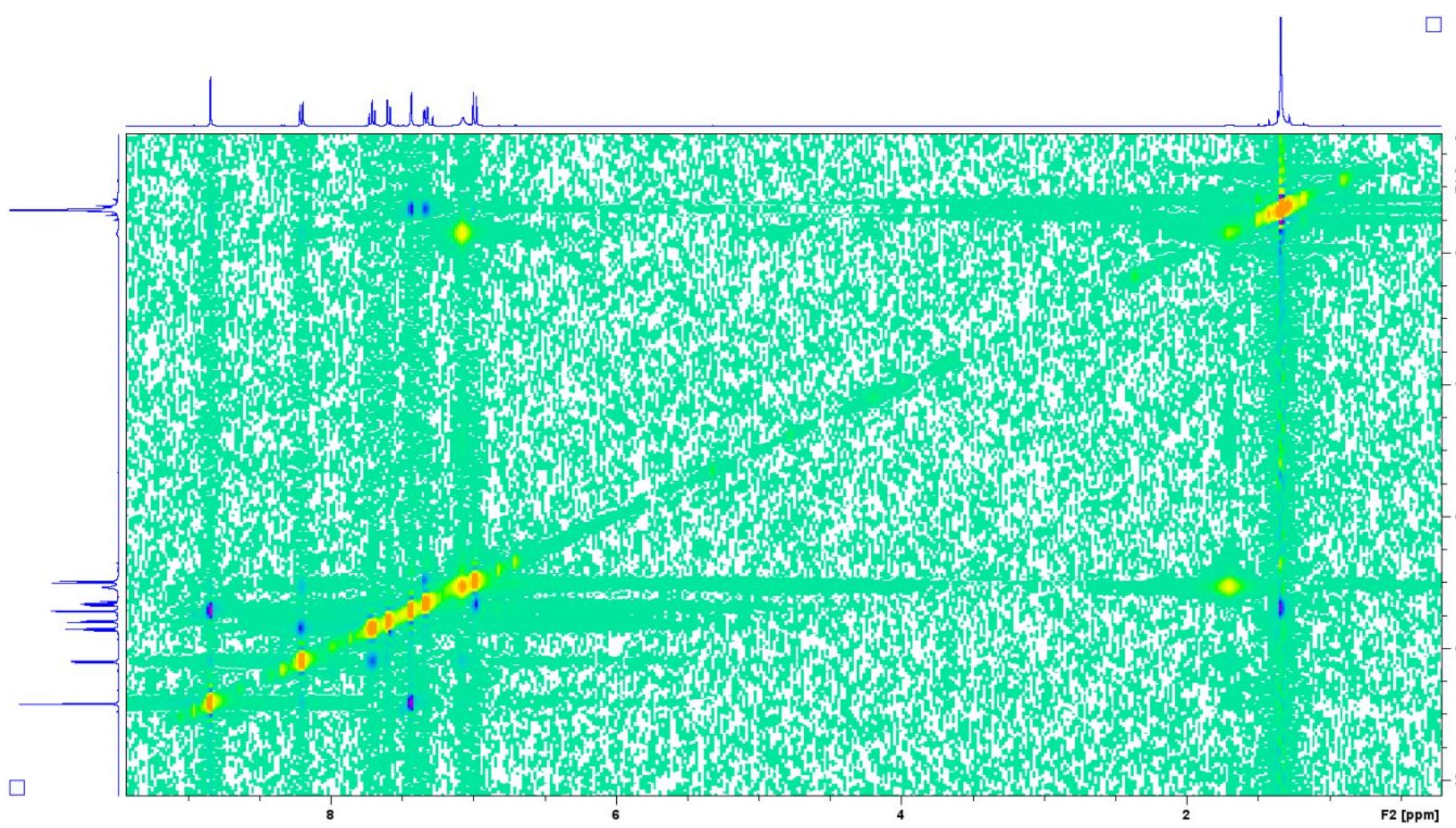

Figure S5. NOESY spectrum of BPTP in $CDCl_3$ at room temperature.

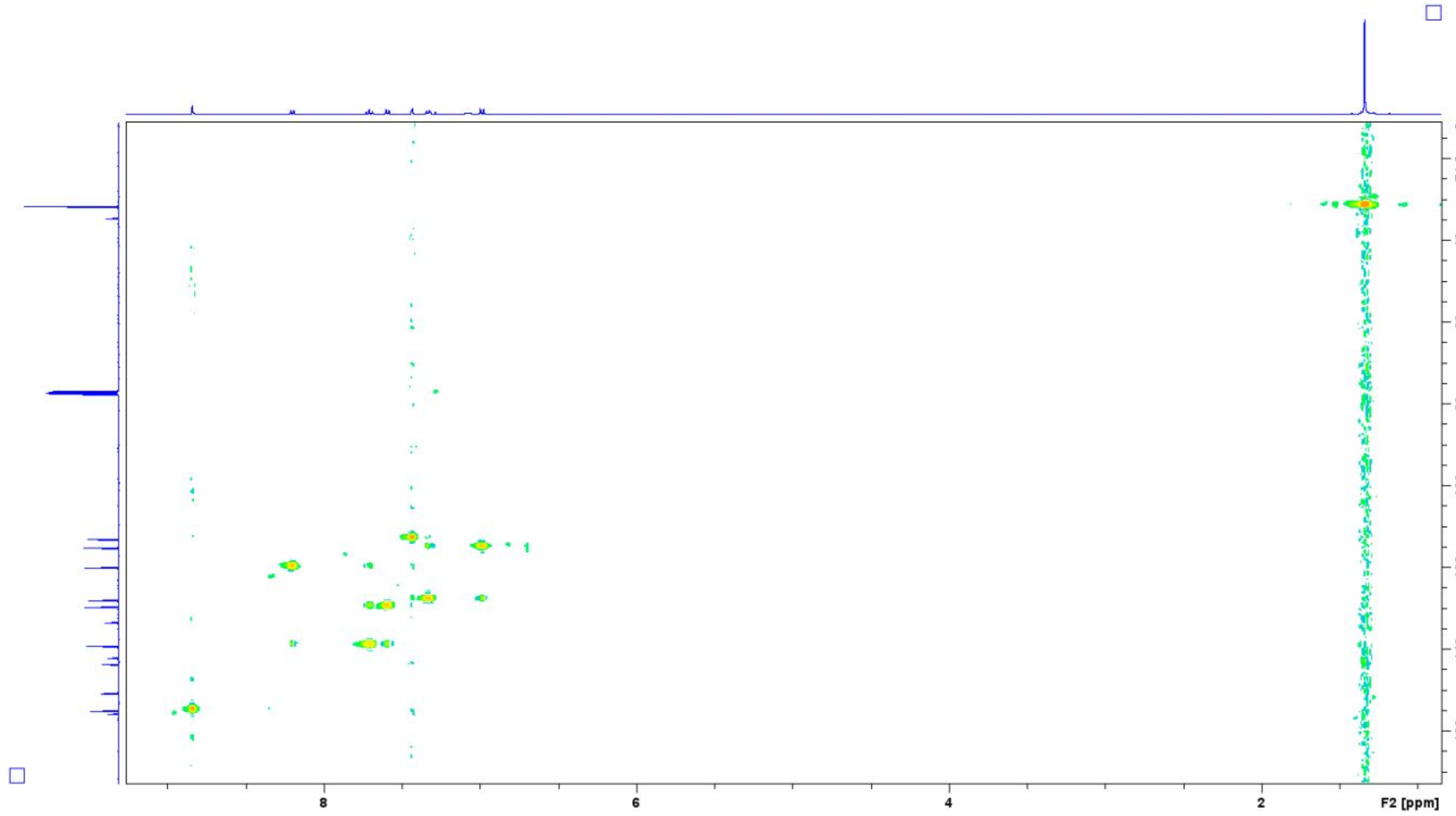

Figure S6. HSQC spectrum of BPTP in $CDCl_3$ at room temperature.

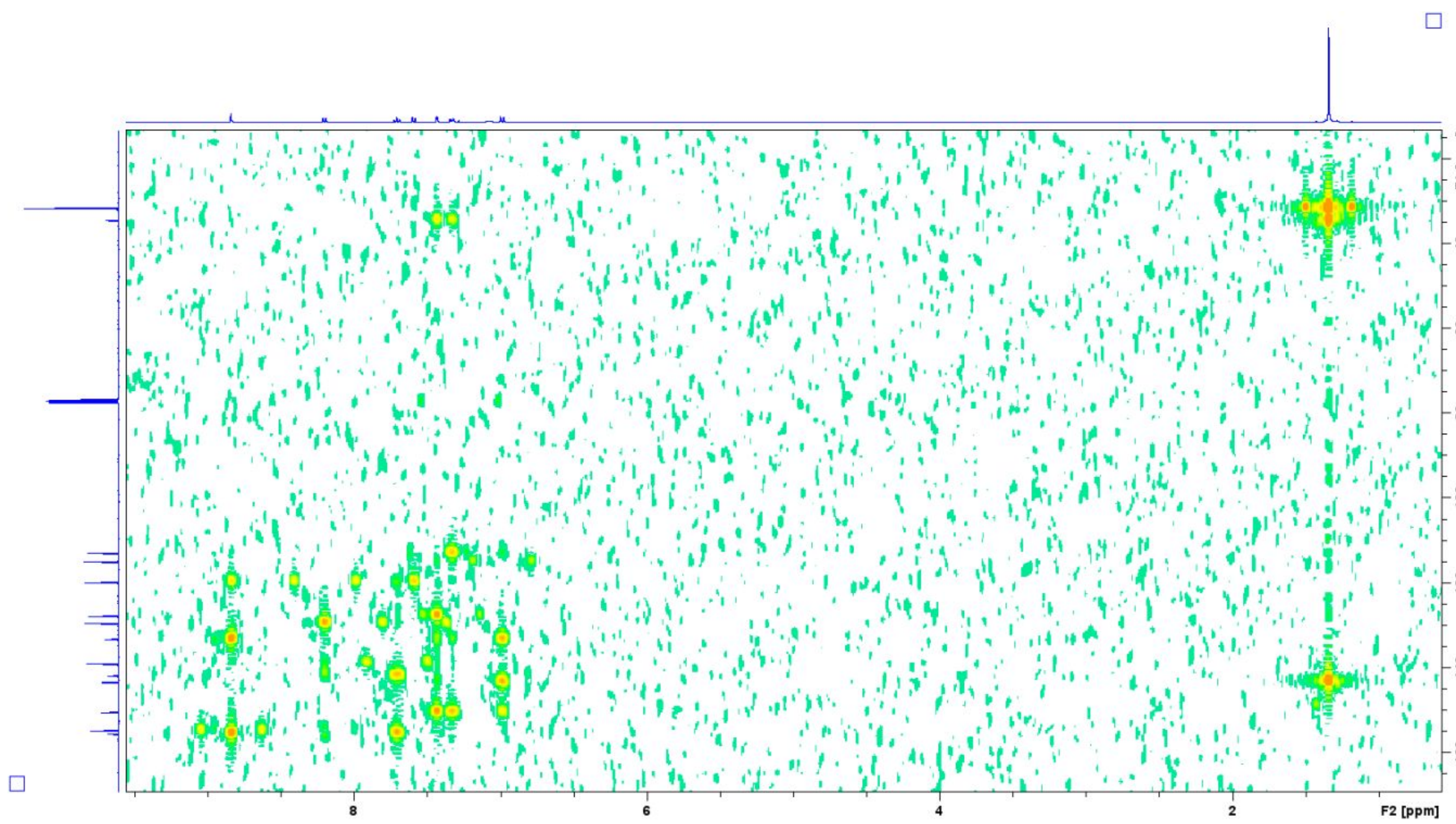

Figure S7. HMBC spectrum of BPTP in $CDCl_3$ at room temperature.

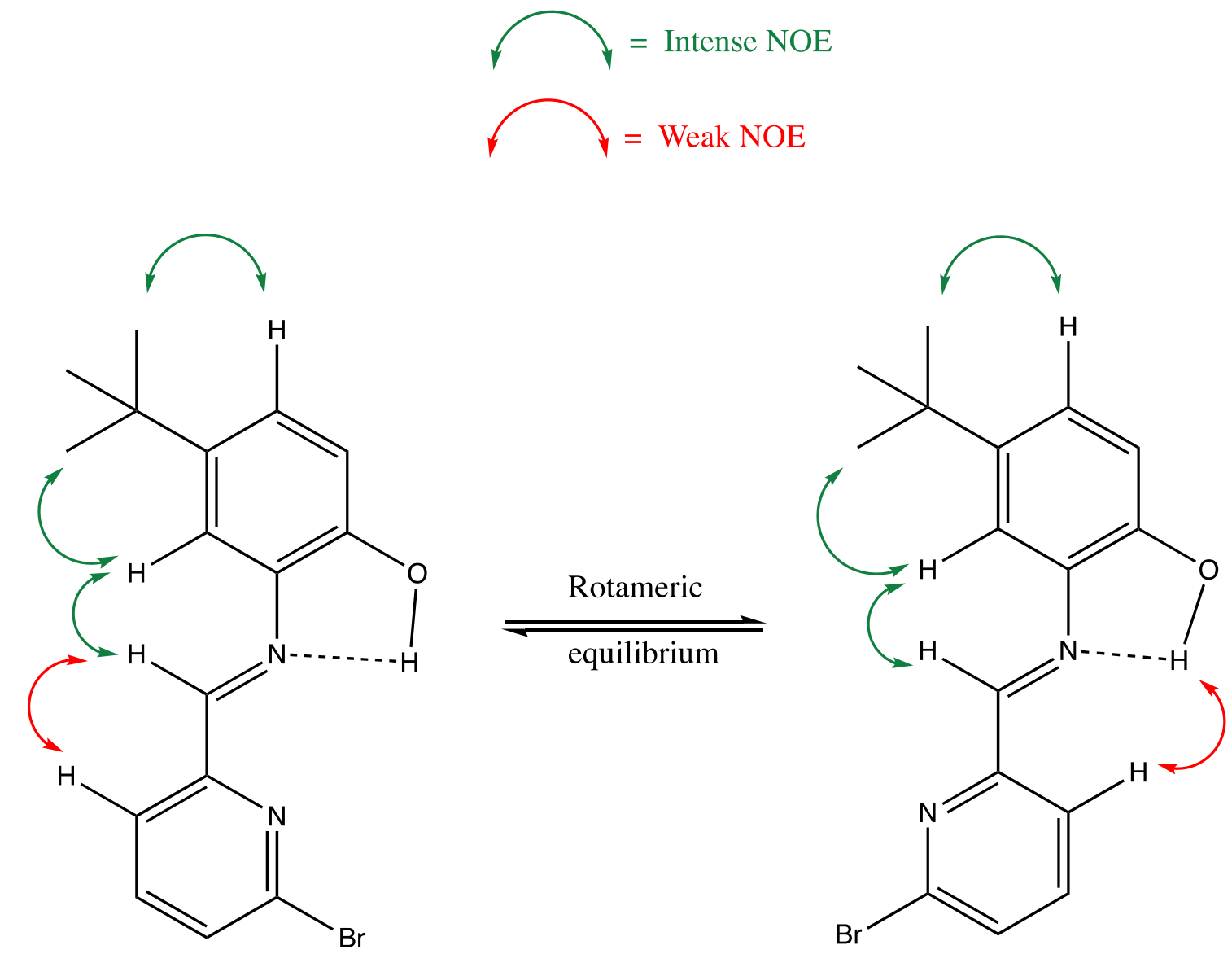

Figure S8. Rotameric equilibrium present in BPTP observed by NOESY spectrum.

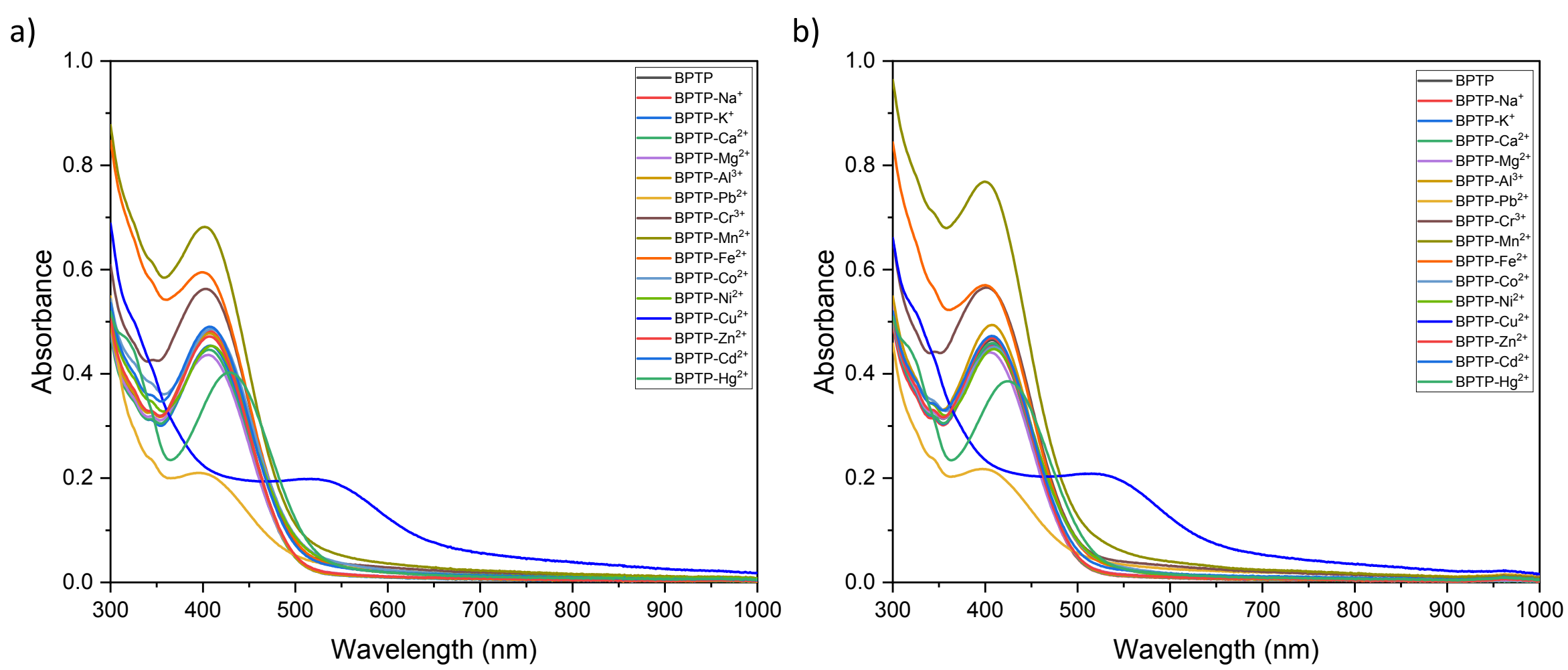

Figure S9. Analytical response of BPTP (100 µM) in the presence of different metal ions (100 µM) in water, a) pH 8 and b) pH 9.

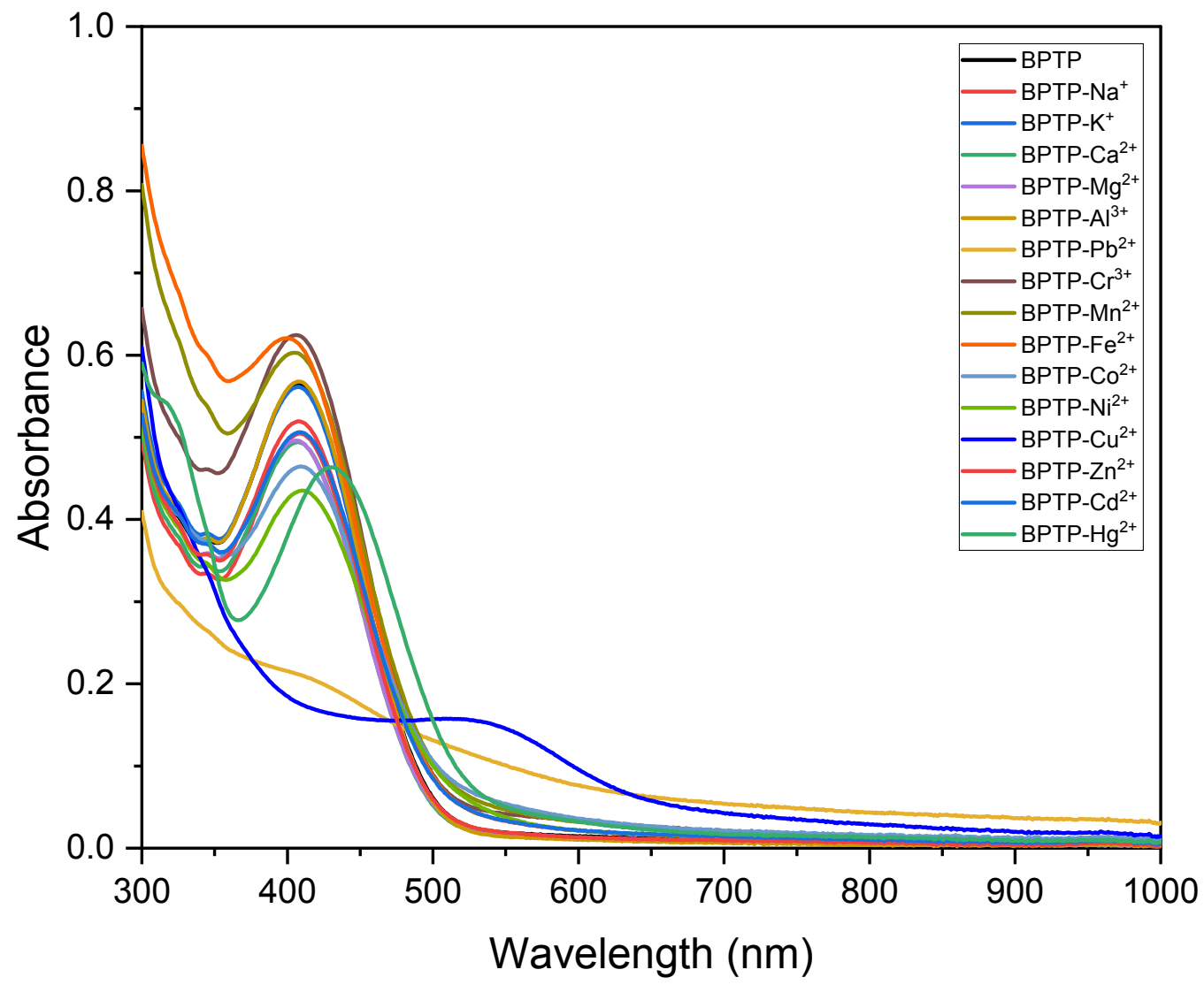

Figure S10. Analytical response of BPTP (100 μM) in the presence of different metal ions (100 μM) in water at pH 6.

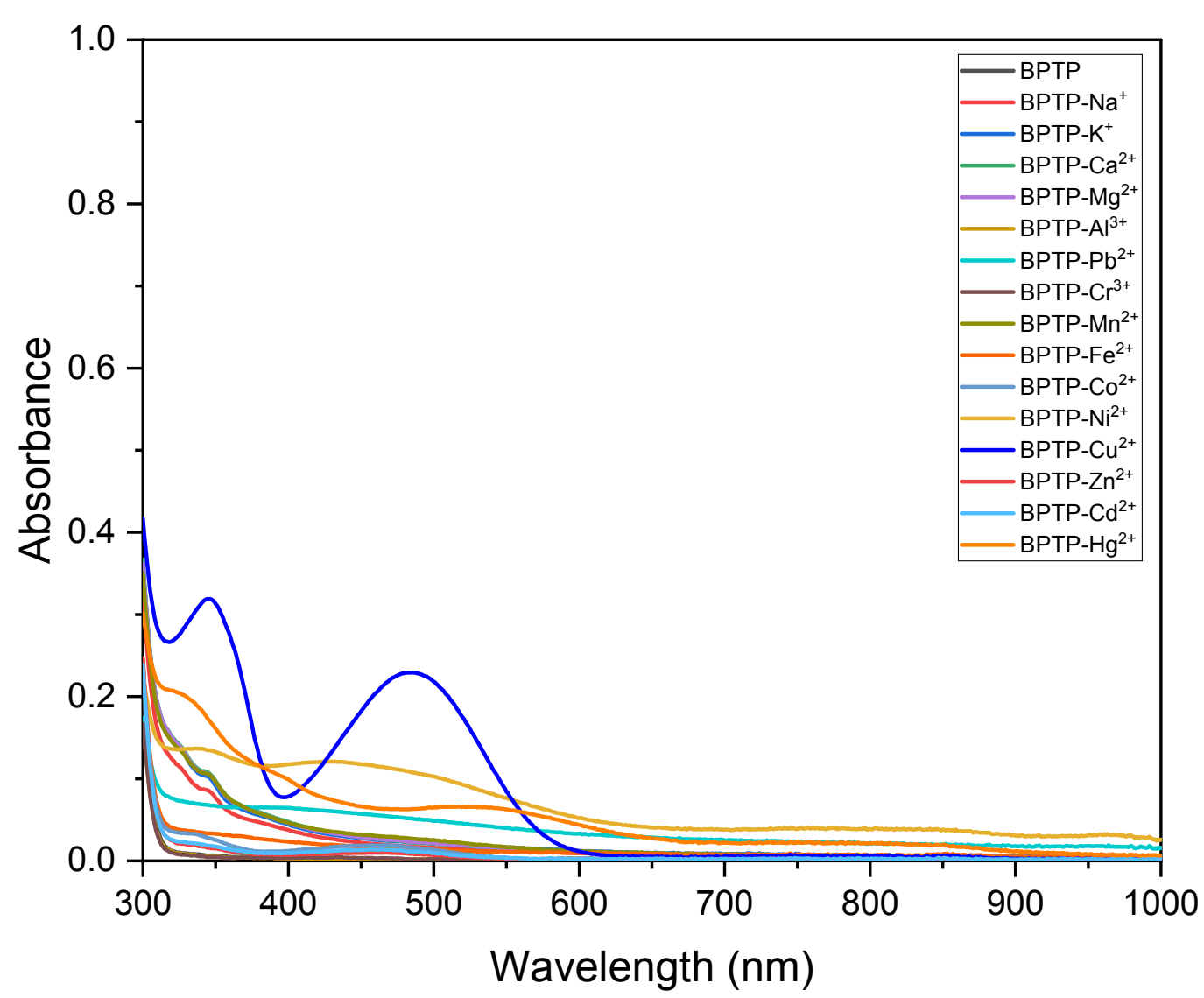

Figure S11. Analytical response of BPTP (100 μM) in the presence of different metal ions (100 μM) in water at pH 5.

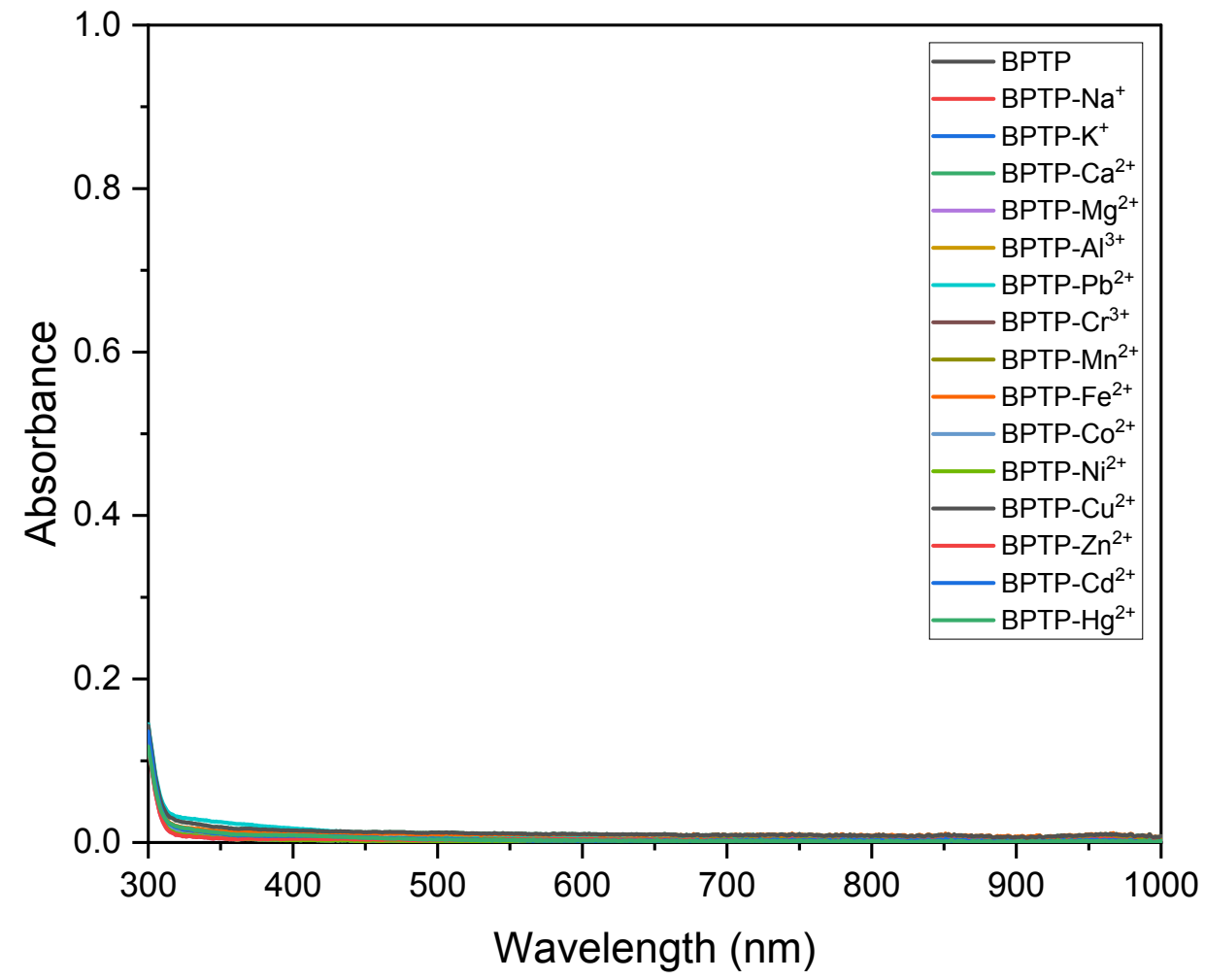

Figure S12. Analytical response of BPTP (100 μM) in the presence of different metal ions (100 μM) in water at pH 4.

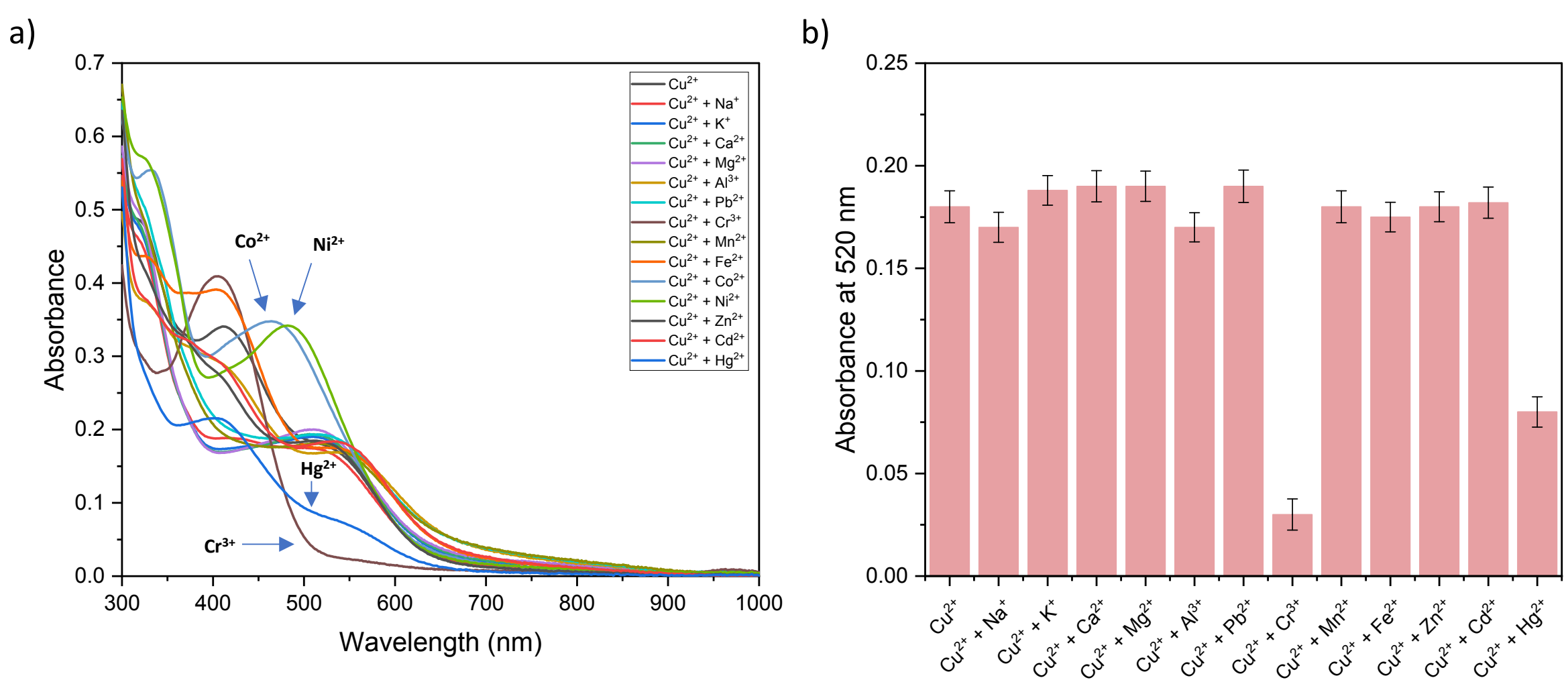

Figure S13. Competitive assay of mixtures of Cu^{2+} (25 μM) and other metal ions (25 μM) in the presence of BPTP (100 μM) in water at pH 7. a) absorption spectra and b) absorbance intensity at 520 nm.

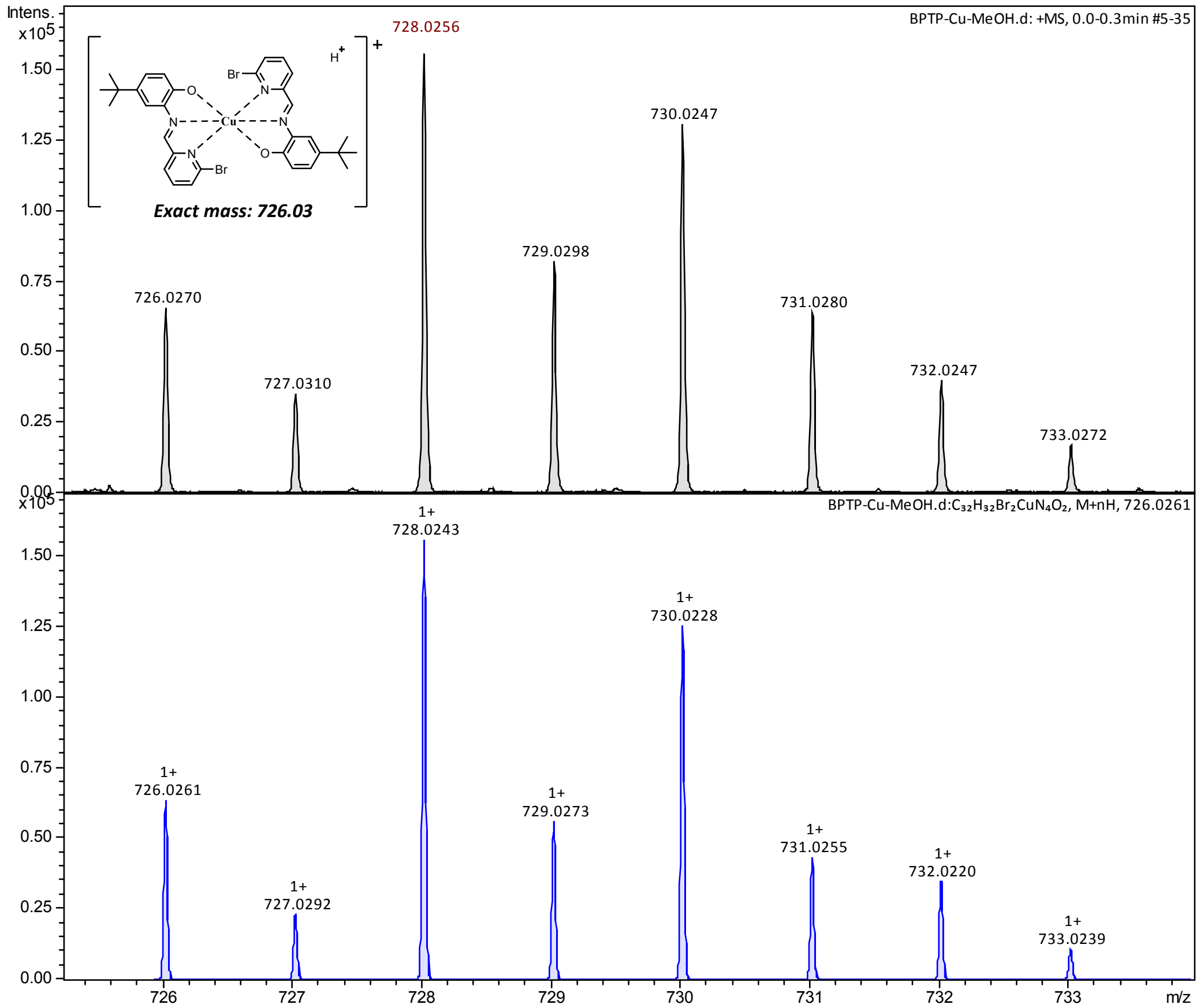

Figure S14. ESI-MS spectra of BPTP-Cu complex (0.5 equiv. of Cu^{2+}): experimental spectra (black line) and theoretical spectra (blue line).

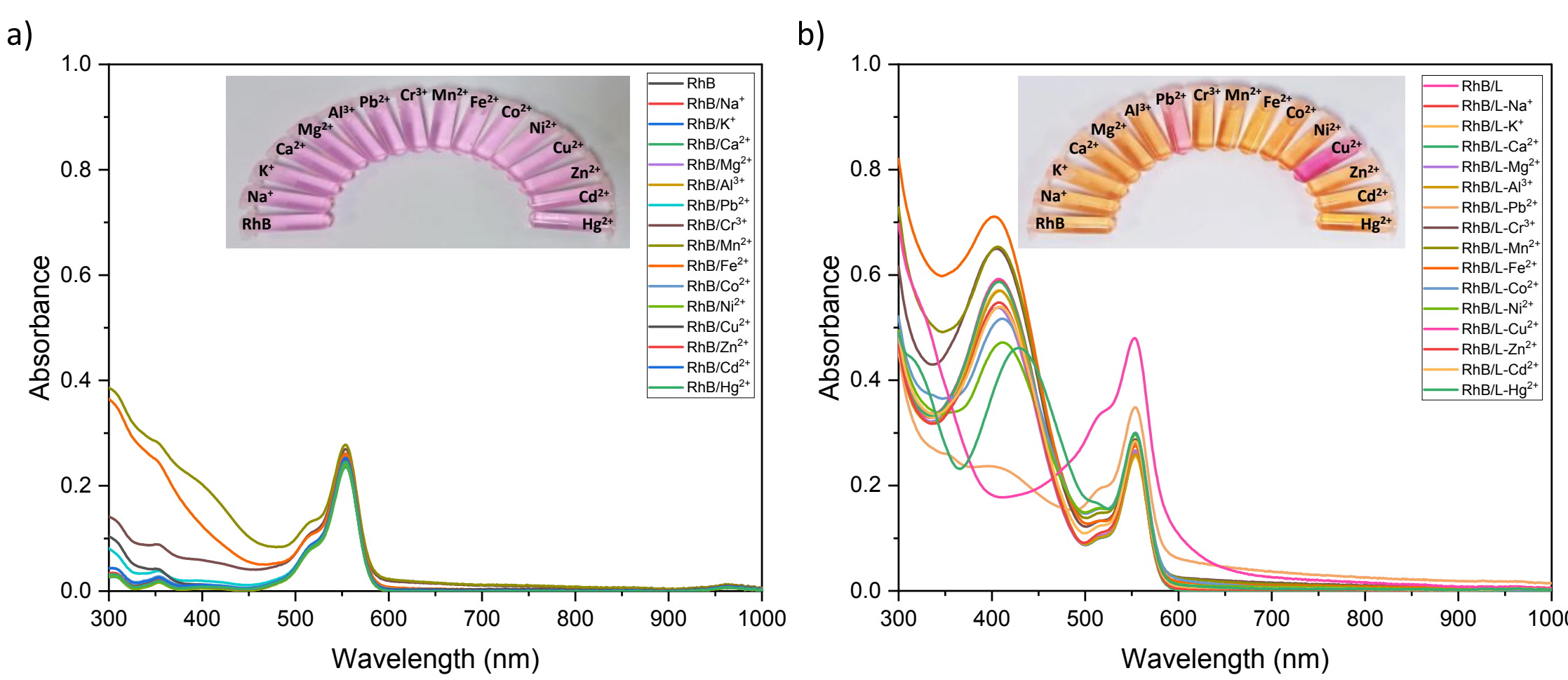

Figure S15. UV-Vis spectra of RhB, in the presence of a) metal ions and b) BPTP-metal complexes (L: BPTP).

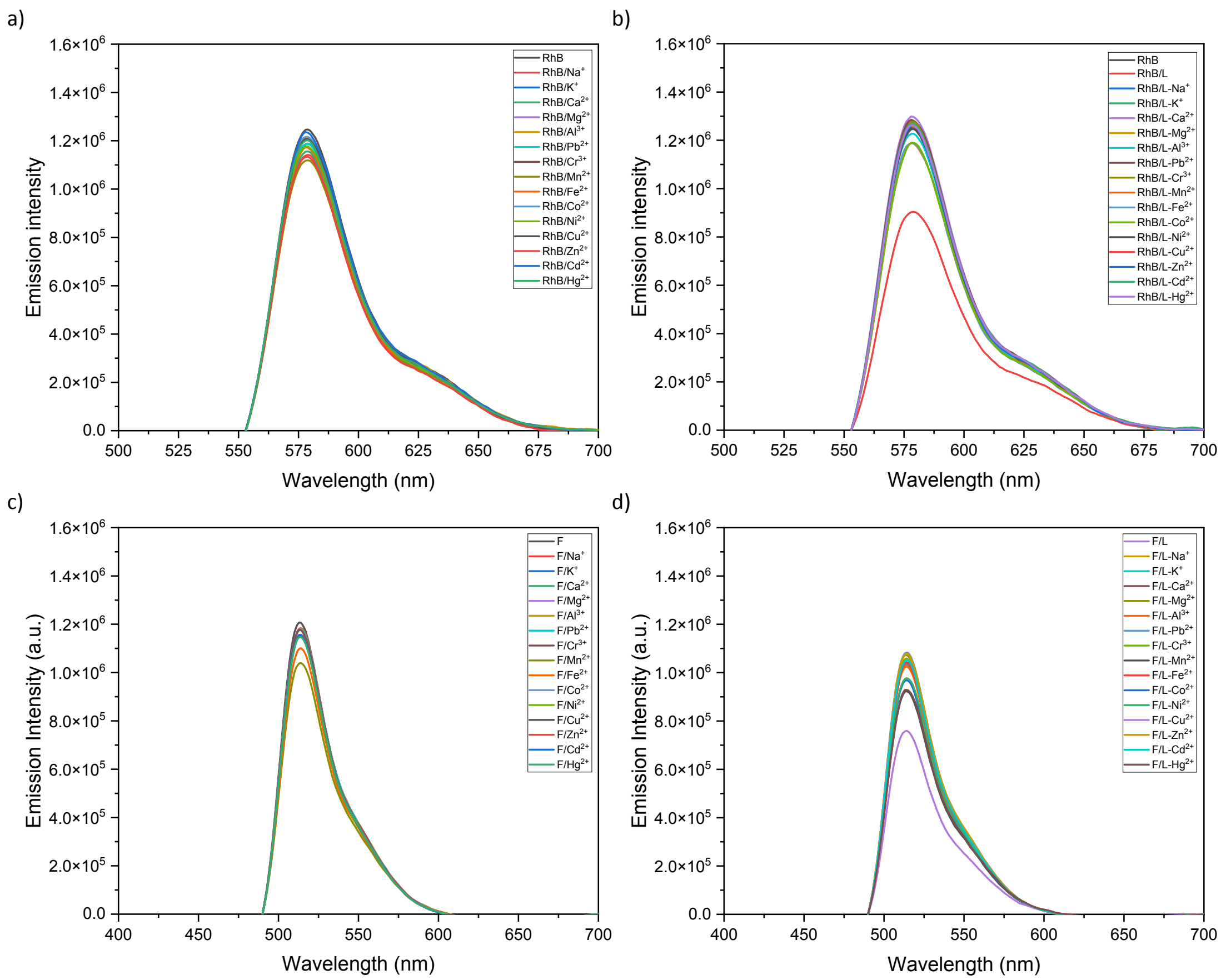

Figure S16. Photoluminescent response of (a, b) rhodamine and (c, d) fluorescein, in the presence of metal ions and BPTP-metal complexes, respectively (L: BPTP).

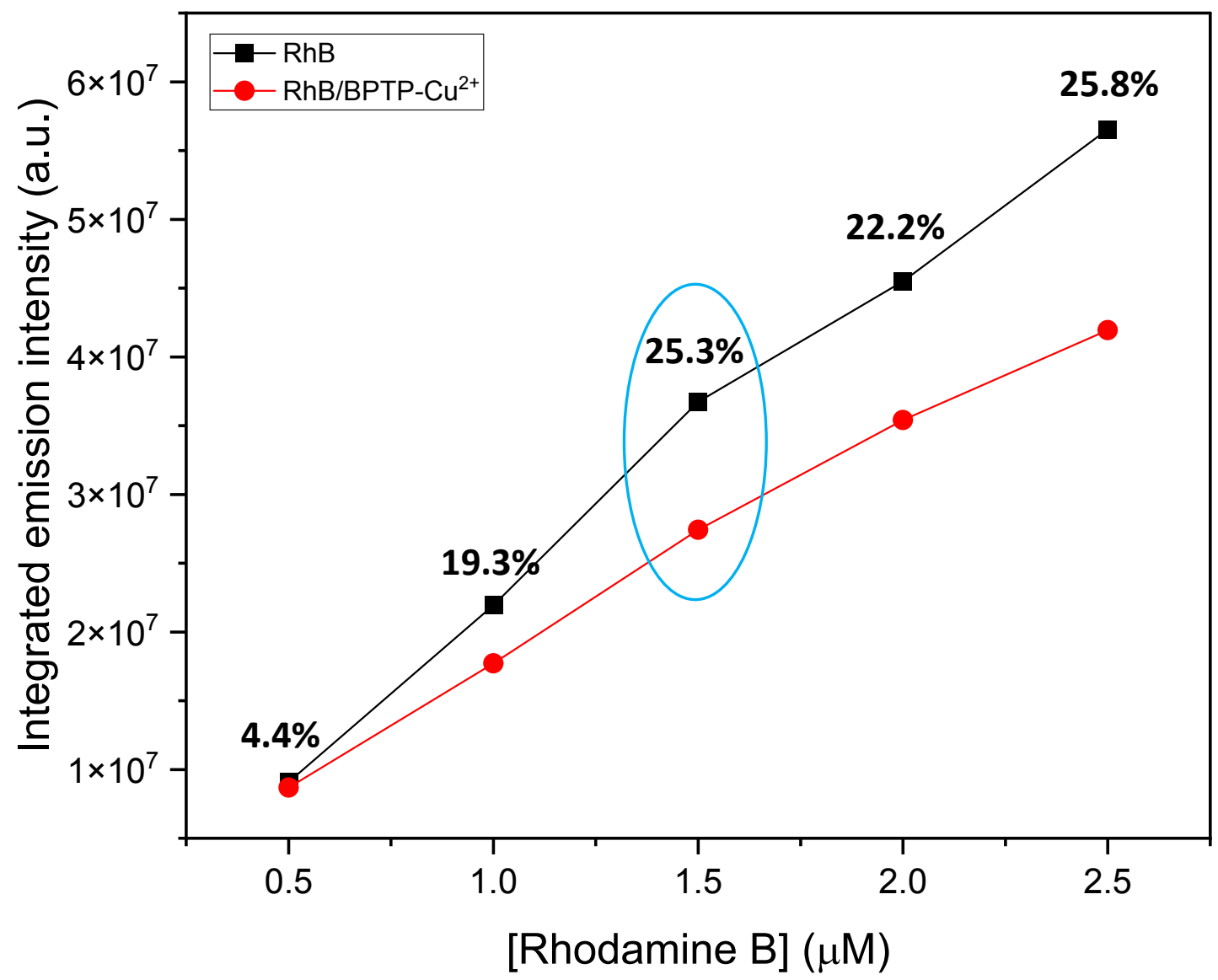

Figure S17. Relative emission intensity of RhB-BPTP-copper (red line) and free RhB (black line).

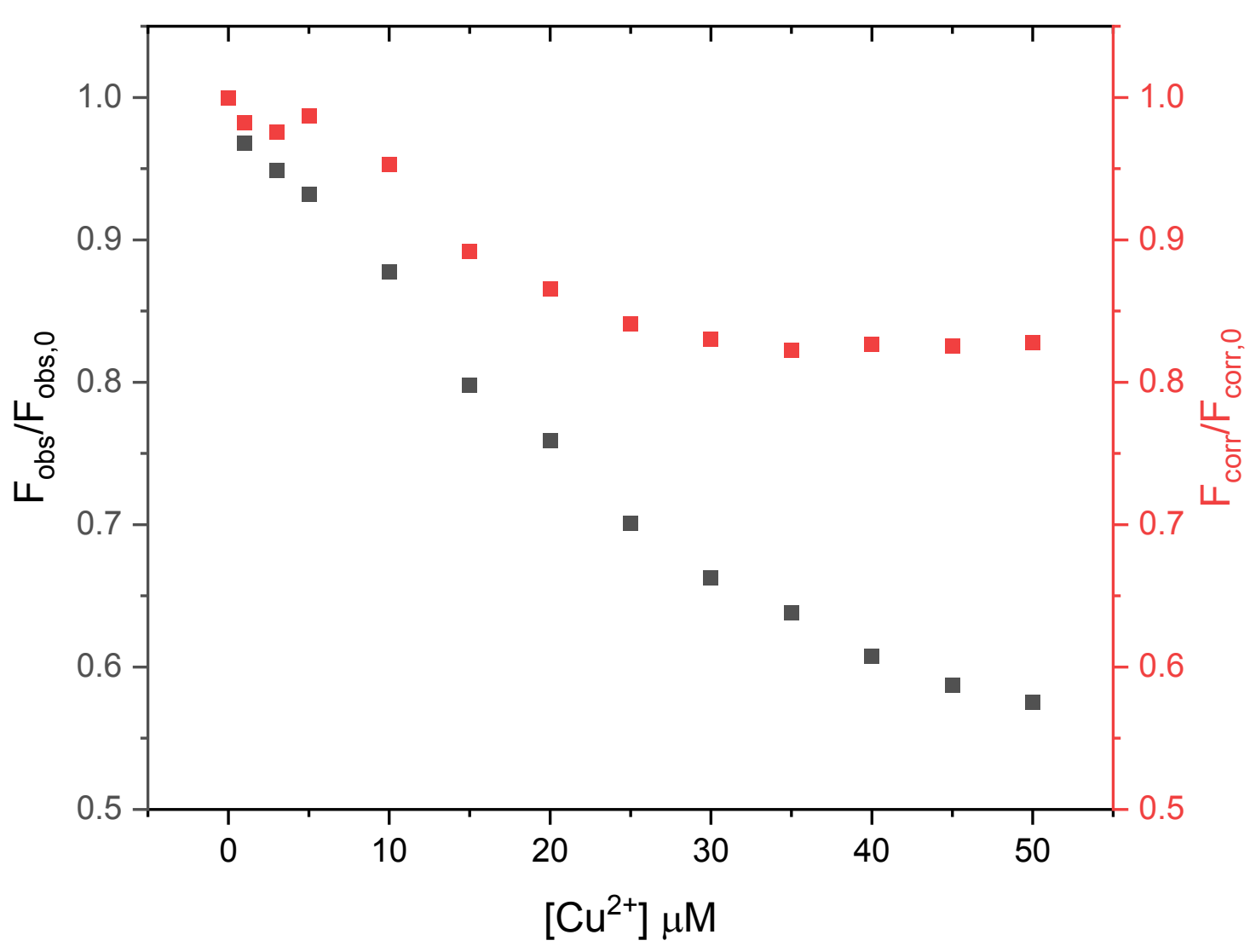

Figure S18. Normalized fluorescence intensity of RhB (F/F_0) as a function of Cu^{2+} concentration before and after inner filter effect correction using the Lakowicz equation.

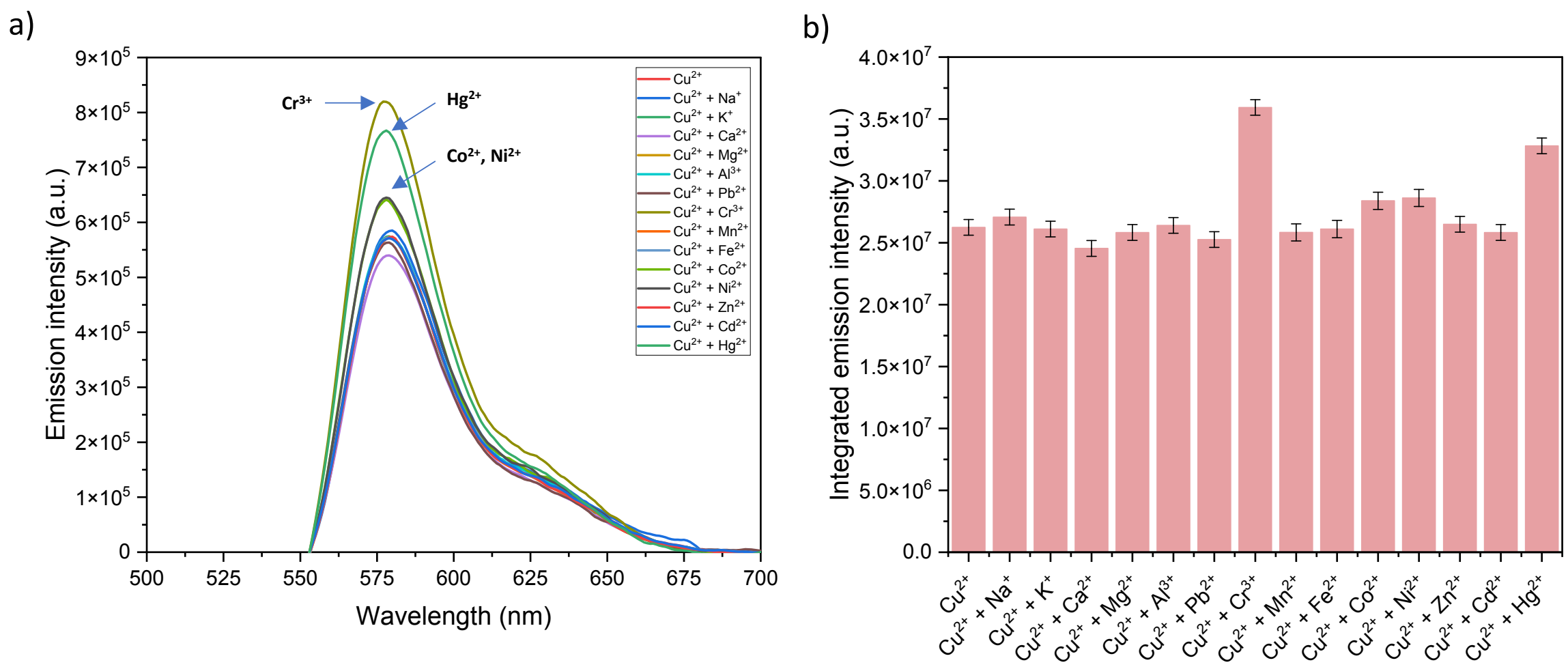

Figure S19. Competitive assay of mixtures of Cu^{2+} (25 μM) and other metal ions (25 μM) in the presence of BPTP (100 μM) and RhB (1.5 μM) in water at pH 7. a) emission spectra and b) integrated emission intensity.

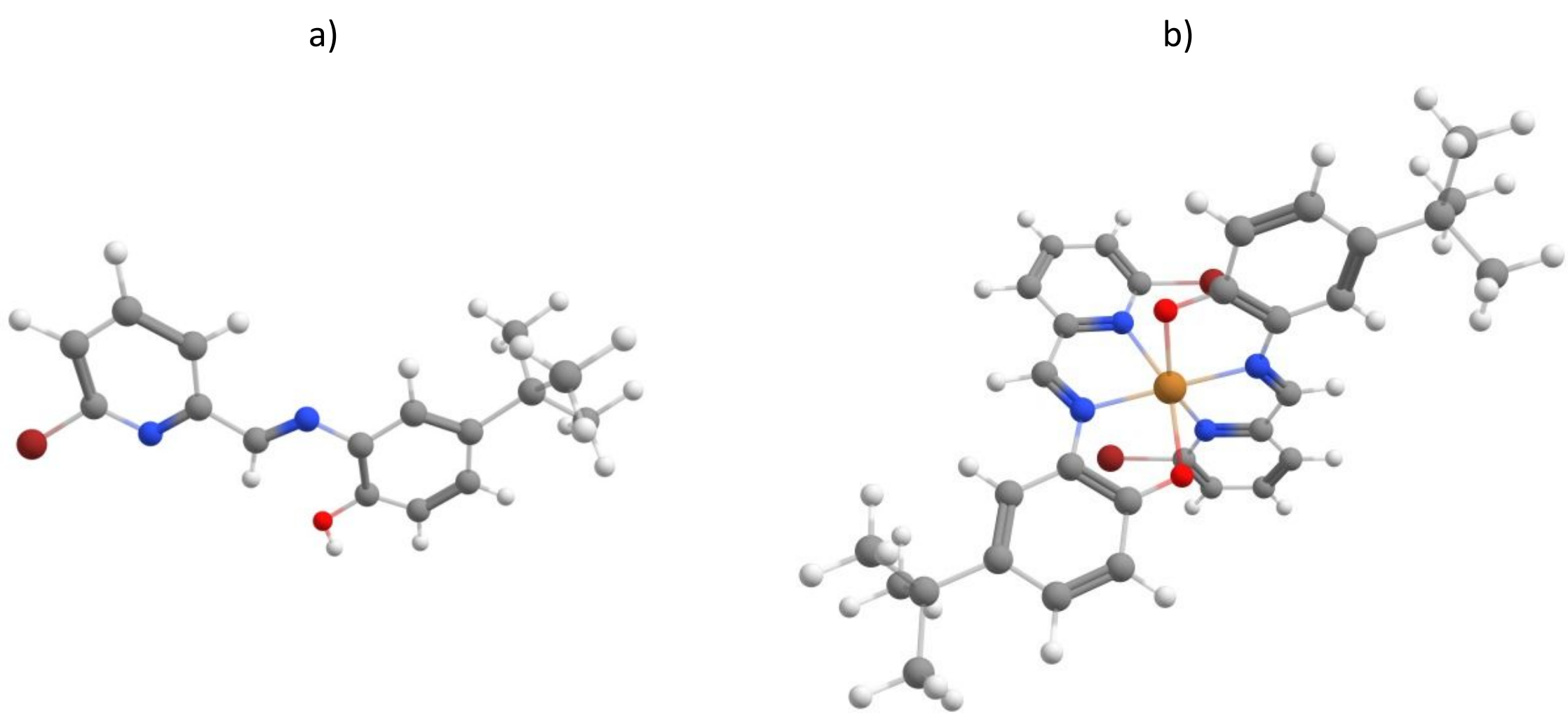

Figure S20. Optimized structure of a) BPTP and b) BPTP-Cu complex.

Table S1. Absorbance values of the RhB/BPTP-Cu^{2+} system at the excitation (550 nm) and emission (580 nm) wavelengths used for the inner filter effect correction.

**[Cu^{2+}] uM**	**A_{exc} (550 nm)**	**A_{em} (580 nm)**
0	0.1234	0.0236
1	0.1315	0.028
3	0.1435	0.0278
5	0.1626	0.0342
10	0.1735	0.0449
15	0.1878	0.0553
20	0.2	0.0615
25	0.2239	0.0817
30	0.2336	0.0865
35	0.2611	0.1067
40	0.2878	0.1271
45	0.3029	0.1397
50	0.3163	0.147

Table S2. Corresponding data to the fluorescent calibration curve in the linear range (0-30 μM) for the BPTP-RhB system.

Equation	y = a + b*x
Plot	B
Weight	Instrumental (=1/ei^2)
Intercept	3.56831E7 ± 177170.95473
Slope	-375033.26566 ± 11289.42453
Residual Sum of Squares	16.02576
Pearson's r	-0.99684
R-Square (COD)	0.9937
Adj. R-Square	0.9928

Table S3. Corresponding data to the colorimetric calibration curve in the linear range (0-30 μM) for the BPTP-RhB system.

Equation	y = a + b*x
Plot	B
Weight	Instrumental (=1/ei^2)
Intercept	0.12228 ± 0.00557
Slope	0.00428 ± 3.43647E-4
Residual Sum of Squares	37.27609
Pearson's r	0.98118
R-Square (COD)	0.96272
Adj. R-Square	0.95651

Table S4. Comparison of recently reported Cu^{2+} chemosensors.

Copper sensors	LOD	Medium	Mode response	Ref.
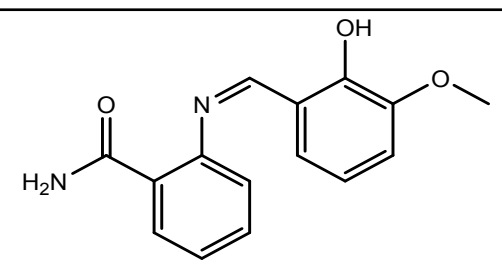	1.57 µM	Bis-Tris buffer/DMSO	Colorimetric	*Coloration Technology*. **2020**, 467, 459–467.
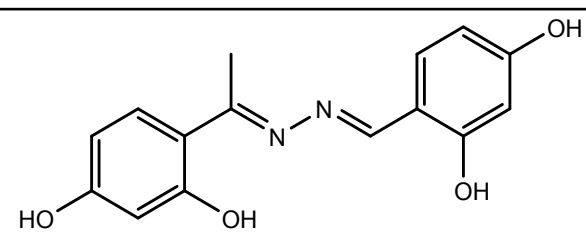	42.09 nM	CH_3CN	Colorimetric	*RSC Adv.,* **2024**, 14, 23083–23094
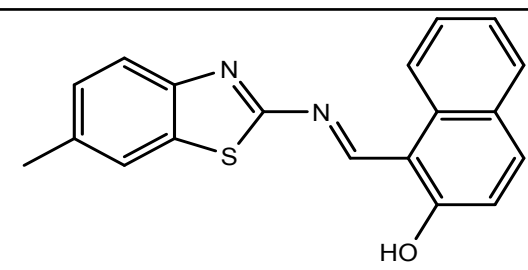	1.6 nM	MeOH-water (98:2 v/v)	Colorimetric	*J. Fluoresc.,* **2025**, 35, 221-236
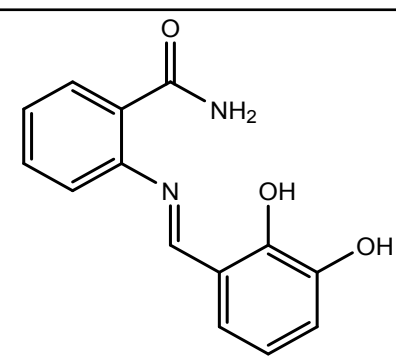	0.9 µM	DMSO: H_2O (1:1, v/v)	Colorimetric	*J. of Fluoresc.,* **2026**, 36, 2633-2642
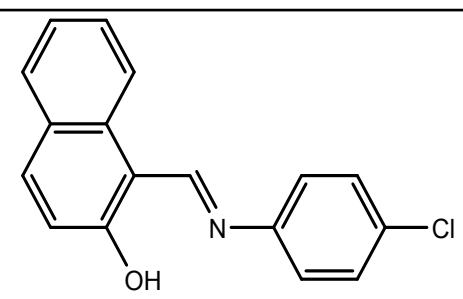	0.049 µM	MeOH-Water (5:95, v/v)	Fluorescent	*Transit. Met. Chem.,* **2026**, 51, 26
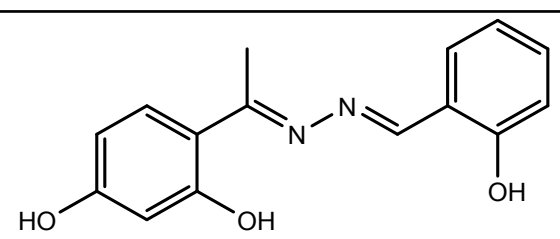	32.2 nM	CH_3CN	Colorimetric	*Discov. Sens.* **2025**, 1, 18.
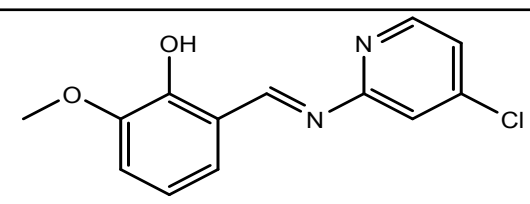	0.17 µM	MeoH-water (80:20 v/v)	Colorimetric	*Int. J. Environ. Anal. Chem*., **2026**, 106, 958-975.
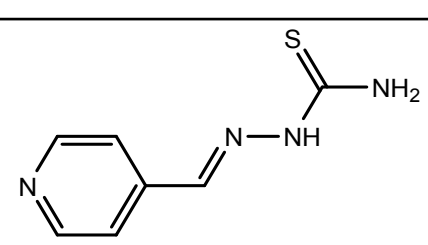	1.7 µM	MeOH-tris-HCl buffer	Colorimetric	Inorg. Chim. Acta., **2020**, 508, 119633.
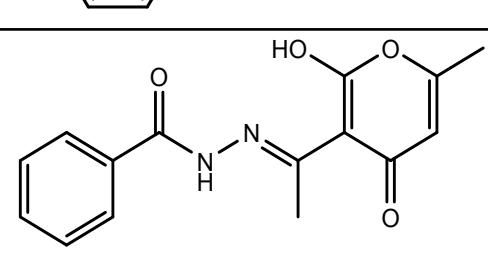	0.156 µM	ethanol: water (8:2 v/v)	Colorimetric	*Microchem. J.*, **2020**, 155, 104705
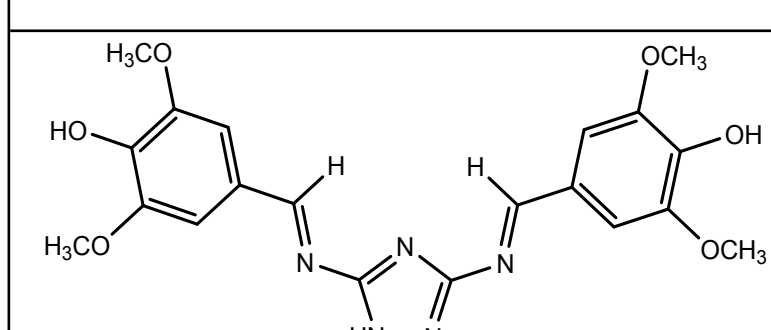	1.2 µM and 3.7 µM	CH_3OH-tris buffer (1:1)	Colorimetric and fluorescent	*RSC Adv.,* **2019**, 9, 25919-25931
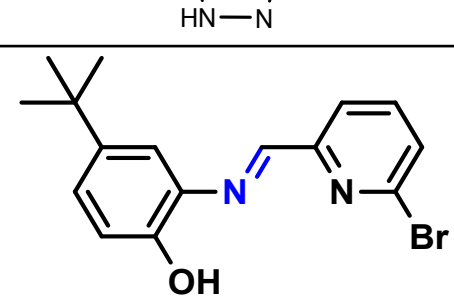	2.1 µM and 1.9 µM	Water	Colorimetric and fluorescent	This work
